# Ultra-rapid near universal TB drug regimen identified via parabolic response surface platform cures mice of both conventional and high susceptibility

**DOI:** 10.1371/journal.pone.0207469

**Published:** 2018-11-14

**Authors:** Bai-Yu Lee, Daniel L. Clemens, Aleidy Silva, Barbara Jane Dillon, Saša Masleša-Galić, Susana Nava, Chih-Ming Ho, Marcus A. Horwitz

**Affiliations:** 1 Division of Infectious Diseases, Department of Medicine, University of California, Los Angeles, California, United States of America; 2 Department of Mechanical and Aerospace Engineering, University of California, Los Angeles, California, United States of America; 3 Department of Bioengineering, University of California, Los Angeles, California, United States of America; Institut de Pharmacologie et de Biologie Structurale, FRANCE

## Abstract

As current treatment of tuberculosis is burdensomely long, provoking non-adherence and drug resistance, effective short-course treatments are needed. Using the output-driven parabolic response surface (PRS) platform, we have identified drug regimens that treat tuberculosis more rapidly in mice than the current Standard Regimen used in humans. We show that PRS Regimen III, comprising clofazimine, SQ109, bedaquiline and pyrazinamide, rapidly sterilizes the lung both in conventionally studied BALB/c mice and in C3HeB/FeJ mice, highly susceptible mice that develop massive necrotic granulomatous lung lesions akin to those in humans, achieving relapse-free cure in only 4 weeks (p<0.0001 versus Standard Regimen). In contrast, the Standard Regimen required 16 weeks to attain lung culture negative status and 20 weeks to achieve relapse-free cure. Thus, PRS Regimen III dramatically cuts by ~80% the time to relapse-free cure in mouse tuberculosis models. PRS Regimen III, with three nonstandard drugs, can potentially treat both drug-sensitive and most drug-resistant tuberculosis.

## Introduction

Tuberculosis (TB) is the leading cause of death worldwide from a single infectious agent. Drug-susceptible TB typically is treated with four first-line drugs, isoniazid (INH), rifampicin (RIF), ethambutol (EMB) and pyrazinamide (PZA), for 8 weeks followed by INH and RIF for 16 weeks or longer as necessary [1]. Poor adherence, drug toxicity, development of drug resistance, and treatment failure often complicate this lengthy treatment course. Approximately 5% of new TB cases are multidrug resistant (MDRTB) with the bacteria resistant to both INH and RIF [2]. Treatment of MDRTB is even more problematic, as treatment regimens typically involve prolonged treatment of a year or more with second and third line drugs with lower efficacy/ toxicity ratios. In 2016, WHO endorsed a shorter MDRTB regimen; however, this 7-drug regimen requires 9–12 months of treatment [3,4]. Even more ominous is extensively drug-resistant TB (XDRTB), resistant to not only INH and RIF but also fluoroquinolones and aminoglycosides. Shorter treatment regimens effective against MDRTB and XDRTB are sorely needed.

We have found that drug-dose inputs (i.e. the specific drugs utilized and their doses) relate to phenotypic outputs (i.e. the effect of the drugs on a biological system) through a parabolic response surface (PRS) [5–7]. Using PRS methodology to determine the optimal drugs and doses to reduce the viability of *M*. *tuberculosis* in infected macrophage cultures (the phenotypic output), we have identified many combinations of TB drugs that are more potent than the Standard Regimen in inhibiting *M*. *tuberculosis* intramacrophage growth [7]. We have shown in a mouse model of pulmonary TB that one of the regimens, PRS Regimen II, comprising clofazimine (CFZ), bedaquiline (BDQ), EMB, and PZA achieves relapse-free cure with only 4 weeks treatment, a dramatic reduction compared with the 16-weeks required for the Standard Regimen [5].

Our PRS based studies indicated that the experimental drug SQ109 interacts with other TB drugs similarly to EMB and that these two drugs are essentially interchangeable [7]. SQ109 is an EMB analogue with a distinct mode of action. Whereas EMB inhibits arabinosyl transferases essential for cell wall synthesis [8–10], SQ109 disrupts the proton motive force, adversely impacting proton motive force-dependent transporters, including MmpL3, which transports trehalose monomycolate across the inner membrane [11–13].

As PRS methodology indicated that SQ109 and EMB are interchangeable, despite differences in their mechanism of action, we hypothesized that replacing EMB with SQ109 would result in a regimen (PRS Regimen III) that retained superior potency against TB. If so, since three of the four drugs in this regimen (CFZ, BDQ, SQ109) are novel non-first line TB drugs, PRS Regimen III is potentially a universal regimen for drug-sensitive TB, MDRTB (as it omits both INH and RIF) and XDRTB (as it omits a fluoroquinolone and aminoglycoside).

Herein, we evaluate the treatment efficacy of PRS Regimen III including the time to achieve lung sterilization and relapse-free cure in two different mouse models of pulmonary TB–a BALB/c mouse model, which has been widely used for testing drug treatment efficacy and the C3HeB/FeJ (Kramnik) mouse model, which has increased susceptibility to TB [14]. We show that PRS Regimen III is as effective as PRS Regimen II. Treatment with either PRS Regimen II or PRS Regimen III sterilizes the lung of *M*. *tuberculosis* within 5 weeks in BALB/c mice and takes only 4 weeks to achieve relapse-free cure. In the same studies, the Standard Regimen of first-line TB drugs requires 16 weeks to sterilize the lungs and 20 weeks to achieve relapse-free cure. Thus, compared with the Standard Regimen, PRS Regimens II and III reduce time to cure by 75–80% in the BALB/c mice. Remarkably, PRS Regimen III achieves relapse-free cure in the same 4-week timeframe in the C3HeB/FeJ mouse TB model, in which disease is manifest by massive necrotic granulomas. Hence, our study show that PRS Regimen III is dramatically more efficacious than the Standard Regimen in these mouse models of TB. As PRS Regimen III includes three nonstandard drugs, it has the potential to be an ultra-short course near universal TB treatment regimen.

## Materials and methods

### Study design

The objective of the study was the assessment of treatment efficacy, including early bactericidal activity, time to sterilization, and time to relapse-free cure, of a universal regimen, PRS Regimen III, in mouse models of pulmonary TB. First in the BALB/c mouse TB model, the optimal *in vivo* doses for PRS Regimen III, containing the 4 drugs CFZ, SO109, BDQ, and PZA, was determined by defining the drug-dose efficacy response surface in *M*. *tuberculosis*-infected mice. Then the early bactericidal activity of the regimen was assessed by assaying the reduction of *M*. *tuberculosis* CFU in the infected mouse lung over a 14-day treatment period.

Subsequently, a 4-week short-term efficacy study was performed to assess the approximate time required to achieve sterilization in the lung and relapse-free cure. Further definitive assessment of treatment efficacy was conducted in a long-term study comparing PRS Regimen III with the Standard Regimen, comprising the first-line TB drugs INH, RIF, EMB, and PZA, and with PRS Regimen II, comprising CFZ, EMB, BDQ, and PZA, for time to lung sterilization and relapse-free cure over a 22 week treatment period; relapse at each time point was assessed 3 months after the final treatment dose by plating the entire lung for evidence of *M*. *tuberculosis* CFU. Last, the treatment efficacy and occurrence of relapse for PRS Regimen III versus the Standard Regimen were assessed in the C3HeB/FeJ mice. For the efficacy studies, a sample size of 5 mice per group was chosen as it provided 80% power to confirm a minimal mean difference of 2 standard deviations or greater between PRS Regimen III and the Standard regimen using the *p* < 0.05 significance criterion. For the relapse studies in BALB/c and C3HeB/FeJ mice, a sample size of ≥ 8 mice per group (almost always 10 mice/group) was used to allow detection of a somewhat lower frequency of recurrence than would be detectable with 5 mice per group.

### Animals

Six to eight-week old female BALB/c mice (Envigo) and C3HeB/FeJ mice (the Jackson Laboratory) were housed 5 per group, in Innovive ventilated caging systems equipped with HEPA-filtered exhaust in our dedicated UCLA animal facility with HEPA-filtered air supply. Mice were inspected daily for adequacy of food, water, bedding and health conditions. After aerosol infection, mice were monitored for signs of illness (ruffled fur, decreased activity, hunched posture and/or tachypnea) and weight loss. Mice were weighed weekly. Any mouse losing weight or showing signs of illness were weighed daily to insure adherence to the 10% weight loss limit. Any mouse showing clinical signs of illness with a weight loss of >10% of its initial weight was promptly euthanized by hypercarbia using a gradual filling method with a flow rate for 20% volume displacement per min (or a flow rate of 1.5 liters/min for a cage size of 7.5” x 11.75” x 5”). Mice were maintained under CO_2_ flow for at least one minute after respiratory arrest. Cervical dislocation was performed as a confirmatory euthanasia method prior to necropsy. All animal studies were approved by and conducted according to the procedures set forth by the UCLA Animal Research Committee (ARC # 1998–140).

### Aerosol infection

*M*. *tuberculosis* strain Erdman (ATCC 35801) was harvested from infected outbred guinea pig spleens 3 weeks after aerosol infection. Spleens were homogenized in 7H9 medium and cultured on 7H11 agar without antibiotics. Bacteria were harvested from plates into 7H9 media containing 10% glycerol and were subjected to gentle sonication in a water bath to obtain a single bacterial suspension. The sonicated homogenate was allowed to settle for 1 hour. The unsettled suspension was aspirated from above the settled pellet and frozen in 1 ml aliquots at -80°C.

BALB/c mice were infected with a 20 ml single-cell suspension of *M*. *tuberculosis* Erdman at a concentration of 1.875 x 10^6^ CFU/ml in a Collison 6-jet nebulizer fixed to a custom-designed Plexiglas aerosolization chamber contained within a Class II A2 biosafety cabinet; a 30 minute exposure delivered ~250 live bacteria to the lungs of each mouse. C3HeB/FeJ mice were infected by aerosol with a 20 ml single-cell suspension containing 2.5 x 10^5^ CFU/ml of *M*. *tuberculosis* Erdman; a 30 minute exposure delivered ~30 live bacteria to the lungs of each mouse (Day 0). At Day 1, two mice were euthanized to determine the number of organisms delivered to the lung. At the end of the pre-treatment period—Day 14 (BALB/c mouse model) or Day 42 (C3HeB/FeJ mouse model)—an additional three mice were euthanized to determine *M*. *tuberculosis* CFU counts in the lung at the start of treatment.

### Antimicrobial drugs and treatment

Treatment was begun two weeks (Day 14), in the case of BALB/c mice, or six weeks (Day 42), in the case of C3HeB/FeJ mice, after aerosol infection. Antibiotics were administrated by oral gavage daily (EBA_14_ study) or 5 days per week (Monday–Friday) (all other efficacy and relapse studies). For treatment with the Standard Regimen, RIF was given first and one hour later the combination of INH, EMB and PZA for the first 8 weeks and then the administration of EMB and PZA was discontinued thereafter. For treatment with PRS Regimens, CFZ was administered one hour after the combination of EMB, PZA and BDQ (PRS Regimen II) or SQ109, PZA and BDQ (PRS Regimen III). CFZ, EMB, INH, PZA, and RIF were purchased from Sigma Aldrich (St. Louis, MO). BDQ was purchased from Asclepia (Belgium). SQ109 was kindly provided by Sequella, Inc. RIF was administered in water and all other drugs were prepared as suspension in 0.15% agarose. Sham-treated mice were given water and then 0.15% agarose suspension by oral gavage. Drug doses administered are shown in Table 1.

**Table 1:** Drugs and drug doses used in the studies.

Treatment	Drug dose (mg/kg)			
**Control TB Drug Regimens**	INH	RIF	EMB	PZA
Standard Regimen	25	10	100	150
Enhanced Standard Regimen	25	10	100	450
**Experimental TB Drug Regimens**	CFZ	EMB	BDQ	PZA
PRS Regimen II	25	100	30	450
	CFZ	SQ109	BDQ	PZA
PRS Regimen III*	25	25	30	450
**Drug-Dose Efficacy Response**^†^	CFZ	SQ109	BDQ	PZA
H/L/L/H	25	2.78	5.6	450
H/L/H/L	25	2.78	50	50
H/H/L/L	25	25	5.6	50
H/H/H/H	25	25	50	450
H/H/H/L	25	25	50	50
H/H/L/H	25	25	5.6	450
H/L/H/H	25	2.78	50	450
H/H/M/M	25	25	16.7	150
H/M/H/M	25	8.25	50	150
H/M/M/H	25	8.25	16.7	450
H/H-M/H/H	25	16.75	50	450
H/M/H/H	25	8.25	50	450

*The optimal doses for drugs in PRS Regimen III, with CFZ, SQ109, BDQ and PZA at 25, 25, 30 and 450 mg per kg, respectively, that were used in animal studies assessing Time to Lung Sterilization and Time to Relapse-free Cure, were determined from the drug-dose efficacy response surface.

^†^The drug-dose efficacy response surface was generated from data on the lung burden of *M*. *tuberculosis* in mice treated with dose variations of the regimen, as follows: H, high dose; H-M, high-middle dose; M, middle dose; L, low dose.

Optimization of *in vivo* drug doses by defining the drug-dose efficacy response surface. PRS methodology follows from our observations that the drug—dose response surface is smooth and can be described accurately by a second-order quadratic algebraic equation expressed as:

$$y=\beta_{0}+\beta_{1}x_{1}+\cdots +\beta_{n}x_{n}+\beta_{12}x_{1}x_{2}+\cdots +\beta_{mn}x_{m}x_{n}+\beta_{11}x_{1}{}^{2}+\cdots +\beta_{nn}x_{n}{}^{2}$$

where y represents the desired output (i.e. log_10_ CFU in this study), x_n_ represents the n^th^ drug dosage, β_0_ represents the intercept term, β_n_ represents the single-drug coefficient of the n^th^ drug, β_mn_ represents the drug-drug interaction coefficient between the m^th^ and n^th^ drugs, and β_nn_ represents the quadratic coefficient for the n^th^ drug. Twelve groups of mice were evaluated to solve the coefficients of this equation. The mice were infected with *M*. *tuberculosis* and treated by oral gavage 5 days per week for 3 weeks with a combination of 4 drugs (CFZ, SQ109, BDQ and PZA) in PRS Regimen III; mice in the different groups were treated with different dose ratios of SQ109, BDQ and PZA (Table 1). The lung log_10_ CFU data for the 12 groups at the end of three weeks treatment were analyzed using MATLAB (S1 Code) to obtain the fitted second-order quadratic algebraic equation (Eq 1) with drug doses as the predictor variables and lung log_10_ CFU counts as the response variables. Stepwise regression was then initialized with first-order drug terms, drug-drug interaction terms, and second-order drug terms, using the corresponding estimated coefficients. The stepwise regression results provide the relative contribution of each term to the desired output. The predictive power of the generated equation was calculated with adjusted R^2^ and with correlation coefficients in order to verify PRS optimization. Correlation coefficients were calculated from experimental output values and from projected output values for the respective drug combinations.

### Assessment of EBA_14_ and Time to Lung Sterilization

Mice were euthanized after oral gavage daily for 14 days (EBA_14_ study) or 5 days (Monday-Friday) per week for a duration of 2–20 weeks (Time to Lung Sterilization studies). Lungs were harvested and homogenized in 1 ml PBS. Lung homogenates from mice that were sham-treated or treated with the Standard Regimen or Enhanced Standard Regimen for 5 weeks or less were serially diluted in PBS and spread, 0.1 ml per plate, on Middlebrook 7H11 agar plates containing 0.4% activated charcoal, ampicillin 12.5 μg/ml, amphotericin B 5 μg/ml, and polymyxin B 20–160 U/ml. The entire volume of lung homogenate for mice receiving treatment with PRS Regimens for any period of time or with the Standard Regimen for more than 5 weeks was plated to obtain the total CFU count per lung. Plates were counted after a 4-week incubation period at 37°C, 5% CO_2_−95% air. Activated charcoal at a concentration of 0.4% was used in the Middlebrook 7H11 agar plates to minimize the effect of possible drug carry-over on the bacterial counts [15]. In our efficacy and relapse studies, we confirmed the absence of any significant drug carry-over effect by examining the number of CFU on serially diluted plates. In cases where bacterial colonies were observed on undiluted plates, we observed 10- and 100-fold fewer colonies on the plates that were diluted 10- and 100-fold, respectively. Moreover, for animals for which we observed no CFU in the undiluted homogenate, 10-fold diluted homogenates also showed no bacterial growth, consistent with true sterilization as opposed to a drug carry-over effect.

### Assessment of Time to Relapse-free Cure

Mice were held for 3 months after the last dose of drug treatment and euthanized to determine the number of bacteria in the lung. The entire volume of lung homogenate was plated for assessment of relapse. Relapse was defined as the presence of 1 or more *M*. *tuberculosis* CFU per lung.

### Sample size and statistical analysis

For Time to Lung Sterilization studies, we used 5 mice per experimental group. For Time to Relapse-free Cure studies, we used animal numbers between 8–14 mice per experimental group. Statistical significance of differences in treatment efficacy across groups was assessed using ANOVA with Tukey’s multiple comparison test in GraphPad Prism 7.04. Percent relapse versus treatment time was compared across groups using the log rank test.

## Results

### The PRS platform identifies optimal drug doses of PRS Regimen III *in vivo* in mice

**Fig 1:**
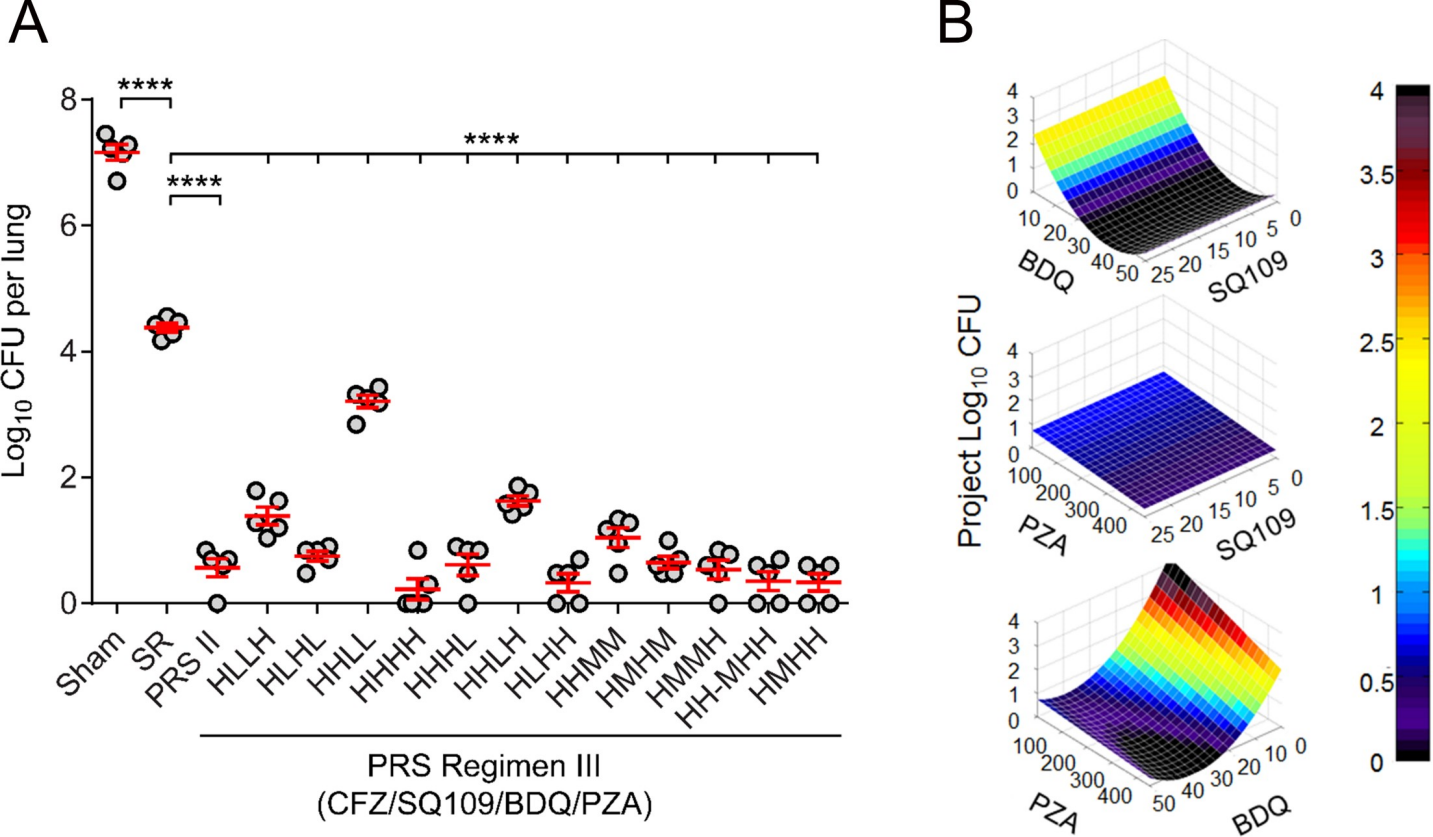
Drug-dose efficacy response surface of PRS Regimen III. (A) Lung log_10_ CFU from BALB/c mice (n = 5 per group) that were sham-treated or treated with the Standard Regimen (SR), PRS Regimen II (PRS II) or PRS Regimen III. Each of the twelve groups of mice treated with PRS Regimen III received the same 4-drug combination with CFZ kept constant at 25 mg/kg and the three other drugs, SQ109, BDQ and PZA, administered at high (H), middle (M), high-middle (H-M) or low (L) dose as shown in Table 1. One mouse each in the groups treated with PRS Regimen III at a dose ratio of HHHH, HLHH, and HMHH had zero CFU in the entire lung; for these mice, a lung CFU count of 1 was assigned for calculation of log_10_ CFU. (B) 3-D surface depicts relationship between doses of two drugs in PRS Regimen III (x and y axes) and projected log_10_ CFU (z axis). One-way ANOVA with Tukey’s multiple comparison test was used in statistical analyses. **** *p* < 0.0001.

We first utilized the PRS platform [5] to determine the optimal doses of drugs in PRS Regimen III *in vivo* in mice. This methodology uses empirical data to derive an equation describing the dose-efficacy response surface, thereby allowing us to calculate the most effective drug dose combination. We infected twelve groups of mice with *M*. *tuberculosis* by aerosol inhalation and treated them 5 days per week for three weeks with the drugs of PRS Regimen III, keeping the dose of CFZ constant at 25 mg/kg and testing the other three drugs, SQ109, PZA, and BDQ, at 4 different dose levels—high, high-middle, middle, or low (Table 1). For each drug, the high dose level was the highest dose used in the literature and the high-middle, middle and low doses were 2/3^rds^, 1/3^rd^, or 1/9^th^ the high dose, respectively. Included in the same experiment were groups of mice that were sham-treated or treated with the Standard Regimen or PRS Regimen II (Fig 1A). The lung burden of *M*. *tuberculosis* was significantly reduced in all treatment groups in comparison with the sham-treated group (*p* < 0.0001, one-way ANOVA with Tukey’s multiple comparison test). For the twelve groups of mice treated with various dose ratios of PRS Regimen III, colony forming unit (CFU) counts in the lung were reduced by 5.54 to 6.84 logs from the level in the sham-treated animals, a significantly greater reduction than the 2.79 log reduction for the group treated with the Standard Regimen (*p* < 0.0001, one-way ANOVA with Tukey’s multiple comparison test) and comparable to the 6.6 log reduction for the group treated with PRS Regimen II (S1 Table).

We used PRS methodology to fit a parabolic equation by regression analysis to the experimental dataset obtained from the twelve groups of mice, using drug doses as predictor variables and treatment outcomes (lung burdens of *M*. *tuberculosis*) as response variables (see Materials and Methods). The resultant equation for the PRS Regimen III drug-dose response surface is:

$$Log_{10}CFU=4.3058-(0.19691)(BDQ)-(0.0041728)(PZA)+(6.5505\;x\;10^{-5})(BDQ)(PZA)+(0.002508)(BDQ^{2})$$ (Eq 1)

where the drug abbreviation in parenthesis represents the dose of the drug in mg/kg.

The resulting equation has an adjusted R^2^ of 0.968 indicating that the equation accounts for 96.8% of the results variance. Color-coded heat maps depicting interaction between any of the two drugs among SQ109, PZA and BDQ showed that, with CFZ kept constant at 25 mg/kg, a low output (lung burden of bacterial CFU) could be achieved with BDQ between 25 and 50 mg/kg and PZA between 250 and 450 mg/kg (Fig 1B). Based on this drug-dose efficacy response, we selected doses of 25/25/30/450 mg/kg for CFZ/SQ109/BDQ/PZA, respectively, for further testing of PRS Regimen III.

### PRS Regimen III exhibits nearly 3-fold higher Early Bactericidal Activity (EBA) than the Standard Regimen

EBA_14_, the average log_10_ CFU *M*. *tuberculosis* reduction/day/ml of patient sputum after 14 days of treatment, is a key measurement in the evaluation of new TB drug regimens, as it is considered a surrogate for sterilizing activity [16,17]. In a conceptually similar study, we assessed the EBA_14_ in the lung of mice, i.e. the mean daily log_10_ CFU reduction in the total lung burden of *M*. *tuberculosis* after daily treatment for 14 days with the Standard Regimen or PRS Regimens II or III (Table 2). The lung log_10_ CFU count at the start of treatment was 6.13. With sham treatment, bacterial log_10_ CFU increased slightly to 6.61 logs at the end of the treatment period. Treatment with the Standard Regimen reduced CFU in the lung by 1.61 logs with an EBA_14_ of 0.12 log_10_ CFU/day. In comparison, treatment with PRS Regimen II reduced lung CFU by 4.65 logs with an EBA_14_ of 0.33 log_10_ CFU/day (*p* < 0.0001 versus Standard Regimen, one-way ANOVA with Tukey’s multiple comparison test), and PRS Regimen III reduced lung CFU by 4.81 logs with EBA_14_ of 0.34 log_10_ CFU/day (*p* < 0.0001 versus Standard Regimen, one-way ANOVA with Tukey’s multiple comparison test). Thus, both PRS Regimens II and III demonstrated a much stronger early bactericidal activity than the Standard Regimen.

**Table 2:** EBA_14_.

Treatment		Log_10_ CFU (Mean ± SEM)*			EBA_14_^†^
Regimen	Drug	Day 1	Day 14	Day 29	
Sham		2.54 ± 0.07	6.13 ± 0.03	6.61 ± 0.13	
Standard Regimen	RIF/EMB/INH/PZA			5.00 ± 0.00	0.12
PRS Regimen II	CFZ/EMB/BDQ/PZA			1.96 ± 0.08	0.33
PRS Regimen III	CFZ/SQ109/BDQ/PZA			1.80 ± 0.19	0.34

*BALB/c mice were infected with *M*. *tuberculosis* by aerosol on Day 0. Three mice were euthanized on Day 1 to determine the number of bacteria delivered to the lungs. Five mice were euthanized on Day 14 to determine bacterial level in the lung prior to treatment. Starting on Day 14, mice (n = 5 per group) were sham treated or treated with the Standard Regimen, PRS Regimen II or PRS Regimen III daily for 14 days. Mice were euthanized one day after the last treatment (Day 29), and the entire lung was homogenized and plated to determine CFU of *M*. *tuberculosis* in the lung. Data shown are means ± SEM.

^†^EBA_14_ = log_10_ CFU reduction/day averaged over 14 days.

### PRS Regimen III markedly shortens the Time to Lung Sterilization

We next evaluated the time required for the PRS Regimens and the Standard Regimen to sterilize the lungs in the BALB/c model of pulmonary TB. We first performed a short-term 4-week efficacy and relapse study to assess the rapidity with which PRS Regimen III sterilizes the lung compared with other regimens, including the Standard Regimen and an “Enhanced Standard Regimen” in which a higher dose of PZA was administered equivalent to that in the PRS Regimens (S2A Table). Subsequently, a long-term study was conducted to determine Time to Lung Sterilization for PRS Regimens II and III versus the Standard Regimen (S2B Table). In both experiments, BALB/c mice were treated with the various regimens 5 days per week (Monday-Friday) by oral gavage. In the first experiment, bacterial CFU counts in the lungs were determined over a course of 2–4 weeks treatment for all groups, and in the second experiment, lung CFU were assayed over a 3–20 week treatment period depending upon the regimen, with PRS Regimens II and III assayed over 3–6 weeks and the Standard Regimen assayed over 3–20 weeks.

In the short-term experiment, consistent with its strong early bactericidal activity, treatment with PRS Regimen III led to a much greater decrease in bacterial burden in the lung than treatment with the Standard Regimen and the “Enhanced Standard Regimen” at 2 weeks, the earliest time point assessed in the experiment (S1 Fig). The 4-log reduction in lung CFU with PRS Regimen III treatment was significantly greater than the 2.5- and 2.7-log reduction with treatment by the Standard Regimen and Enhanced Standard Regimen, respectively (*p* < 0.0001, two-way ANOVA with Tukey’s multiple comparison test). PRS Regimen III treatment continued to decrease bacterial load in the lung at a rapid rate. By 4 weeks, no viable *M*. *tuberculosis* were detected in the lungs of mice treated with PRS Regimen III (S3A Table). At this time point, three out of 5 mice on PRS Regimen II treatment still had a low number of tubercle bacilli present in their lungs (1–3 bacteria per lung). In contrast, mice treated with the Standard Regimen and Enhanced Standard Regimen had 3.1 and 2.5 log_10_ CFU in their lungs at the 4-week time point. Differences in treatment efficacy between PRS Regimens II and III did not reach statistical significance at any of the three time points assessed.

**Fig 2:**
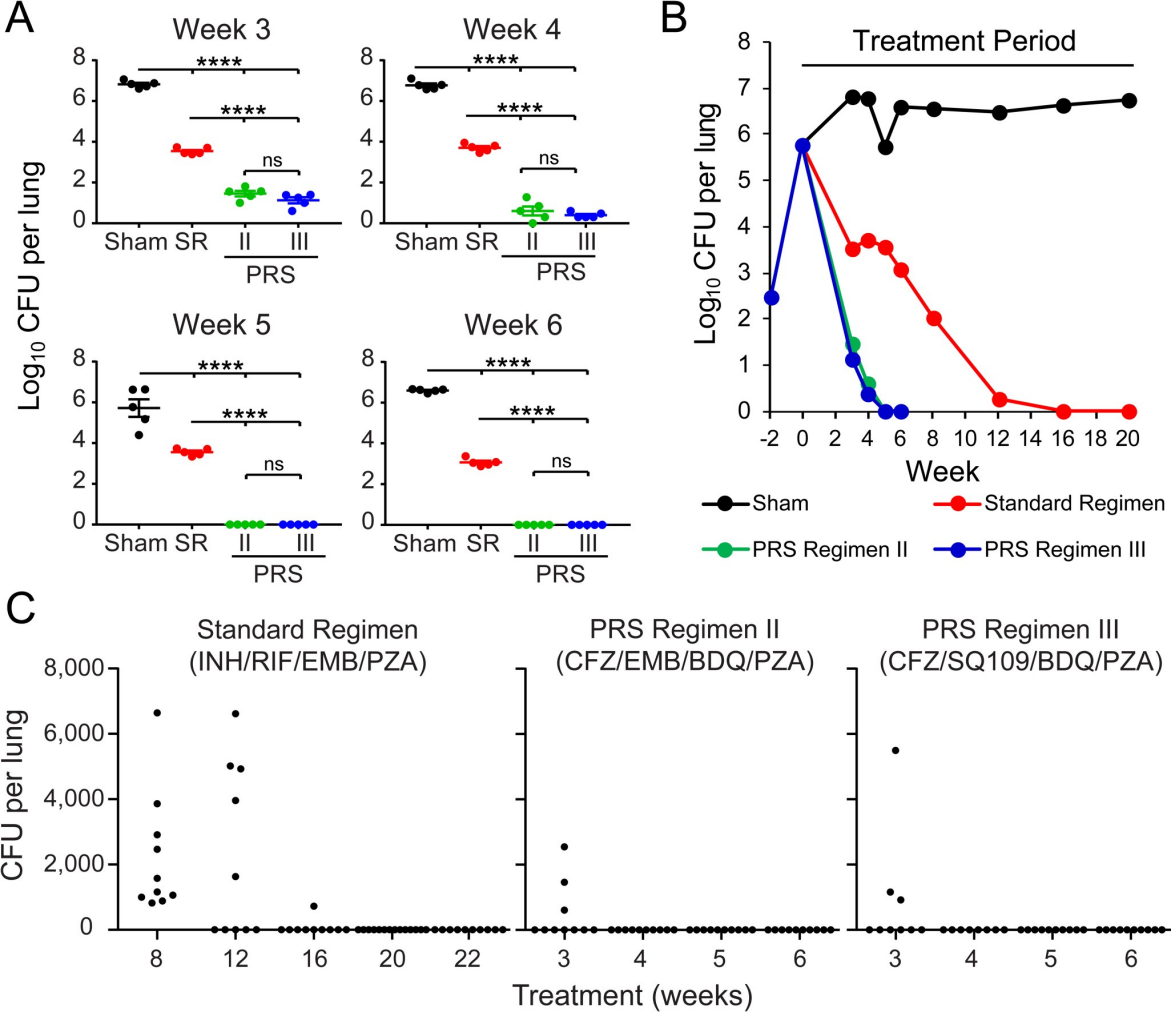
Long-term efficacy (Time to Lung Sterilization) and relapse (Time to Relapse-free Cure) study in BALB/c mice. (A) Lung burden of *M*. *tuberculosis* after 3, 4, 5, and 6 weeks of sham treatment or treatment with the Standard Regimen (SR), PRS Regimen II (PRS II) or PRS Regimen III (PRS III). For mice that had zero CFU in the lungs, a CFU count of 1 was assigned for graphing purposes. Two-way ANOVA with Tukey’s multiple comparison test was used in statistical analyses. **** *p* < 0.0001, ns, not significant (B) Lung burden of *M*. *tuberculosis* over the course of infection and treatment. (C) Relapse. Total number of *M*. *tuberculosis* in the lung of each mouse was determined three months after treatment cessation. Relapse is defined as 1 or more CFU per lung. For all PRS Regimen II groups, PRS Regimen III groups at 5 and 6 weeks, and for the Standard Regimen groups at 8, 12, 16, and 22 weeks, n = 10 mice/group. For the PRS Regimen III group at 3 and 4 weeks, n = 9 and n = 8 mice/group, respectively, and for the Standard Regimen group at 20 weeks, n = 14 mice/group. (*p* <0.0001, PRS Regimen III or PRS Regimen II versus Standard Regimen, log rank test).

**Fig 3:**
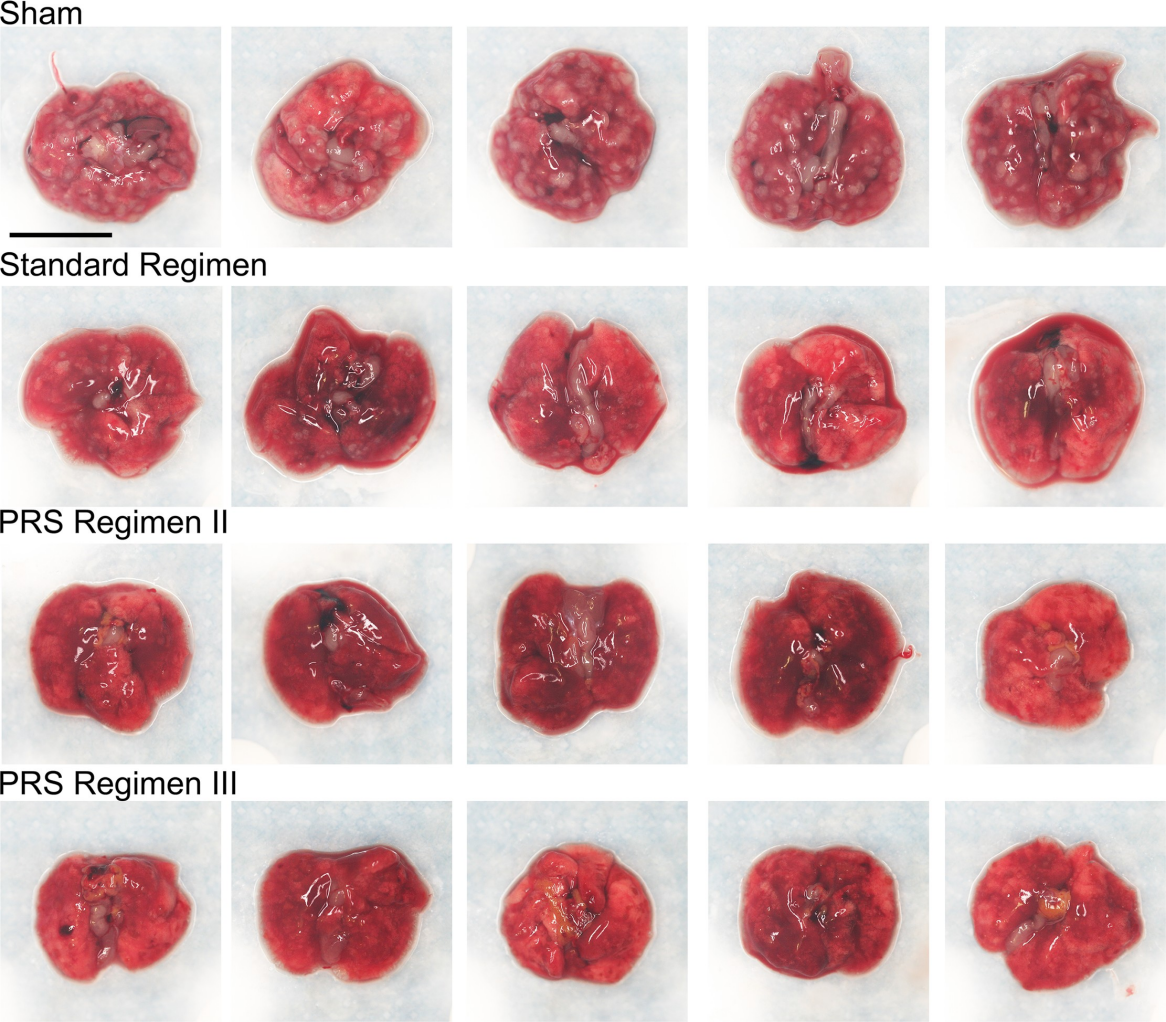
Lung gross pathology in BALB/c mice upon completion of 4 weeks of treatment. Mice (n = 5 per group) were infected with *M*. *tuberculosis* by aerosol and starting two weeks later, they were either sham-treated or treated with the Standard Regimen (INH/RIF/EMB/PZA at 25/10/100/150 mg/kg), PRS Regimen II (CFZ/BDQ/EMB/PZA at 25/30/100/450 mg/kg) or PRS Regimen III (CFZ/BDQ/SQ109/PZA at 25/30/25/450 mg/kg) 5 days per week for 4 weeks. Their lungs and surface granulomas were photographed for inspection. Scale bar (upper left panel), 1 cm.

In the long-term experiment, a rapid reduction in lung CFU was observed again for both PRS Regimens II and III (Fig 2A and 2B). Whereas the bacterial burden in the lungs of sham-treated mice was high throughout the course of the experiment with an average of 6.8 log_10_ CFU at 4 weeks, treatment of mice with PRS Regimens II and III reduced the bacterial burden in the lungs to very low numbers at 4 weeks, ranging between 0–19 and 2–4 organisms per lung for PRS Regimens II and III, respectively (S4A Table). By 5 weeks, the lungs of mice treated with PRS Regimen III were sterilized. At this time point, four mice treated with PRS Regimen II had zero CFU and one mouse had a single CFU in the lung. At 6 weeks, there were no detectable CFU in any of the lungs of mice treated with PRS Regimens II or III. On inspection, the lung gross pathology correlated well with the measured bacterial load. Overall, mice that received sham treatment had 30 or more large lesions evenly distributed over the surface of their lungs. Mice treated with the Standard Regimen had 0–10 smaller lesions on the surface of their lungs. Mice treated with PRS Regimens scarcely had any lesions evident on the surface of their lungs (Fig 3 and S2 Fig). In the same study, 16 weeks of treatment with the Standard Regimen were required to attain lung culture negative status (Fig 2B and S4A Table). Thus, within the same experiment, PRS Regimen III reduced the time required to sterilize the lungs from 16 weeks to 5 weeks compared with the Standard Regimen.

### PRS Regimen III markedly reduces the Time to Relapse-free Cure

Time required to achieve relapse-free cure is critical to the evaluation of new short-course treatments. We assessed TB recurrence 3 months after completion of treatment in both the short-term and long-term study, but were only able to compare the PRS Regimens with the Standard Regimen in the long-term study because of the much greater time required for the Standard Regimen to achieve relapse-free cure. In these studies, we homogenized and plated the entire lungs of the mice to obtain absolute CFU counts in the lungs, and relapse was defined as ≥ 1 CFU in the entire lung. In the short-term study, the percentage relapsing after 2, 3, and 4 weeks of PRS Regimen III treatment was 100%, 40%, and 80%, respectively (S1C Fig and S3C Table); however, of the four mice relapsing after 4-weeks treatment with PRS Regimen III, two had only a single CFU in their lungs. As expected, at the 4-week time point, 100% of mice treated with the Standard Regimen and Enhanced Standard Regimen relapsed, and in these mice CFU counts were in the tens of thousands (S1C Fig and S3B Table).

In the long-term study, 44% of mice treated with PRS Regimen III relapsed with 3-weeks treatment, and none with 4-weeks treatment; similarly, 30% of mice treated with PRS Regimen II relapsed with 3-weeks treatment, and none with 4-weeks treatment (Fig 2C and S4C and S4D Table). Thus, relapse-free cure (defined as no relapse in any animals in the group) required 4-weeks treatment with these PRS Regimens. In contrast, all mice treated with the Standard Regimen relapsed at 8 weeks, 20% at 12 weeks, and 10% at 16 weeks; relapse-free cure was not achieved until 20 weeks (Fig 2C and S4B Table). Thus, compared with the Standard Regimen, PRS Regimen III reduced the time required to achieve relapse-free cure by 80%–from 20 weeks to 4 weeks (*p* <0.0001 versus Standard Regimen, log rank test). There was no difference in the time to relapse-free cure by substituting SQ109 (PRS Regimen III) for EMB (PRS Regimen II) as both PRS Regimens reached relapse-free cure after 4 weeks of treatment (*p* <0.0001, PRS Regimen II versus Standard Regimen, log rank test).

### PRS Regimen III is superior to the Standard Regimen in the C3HeB/FeJ mouse model

**Fig 4:**
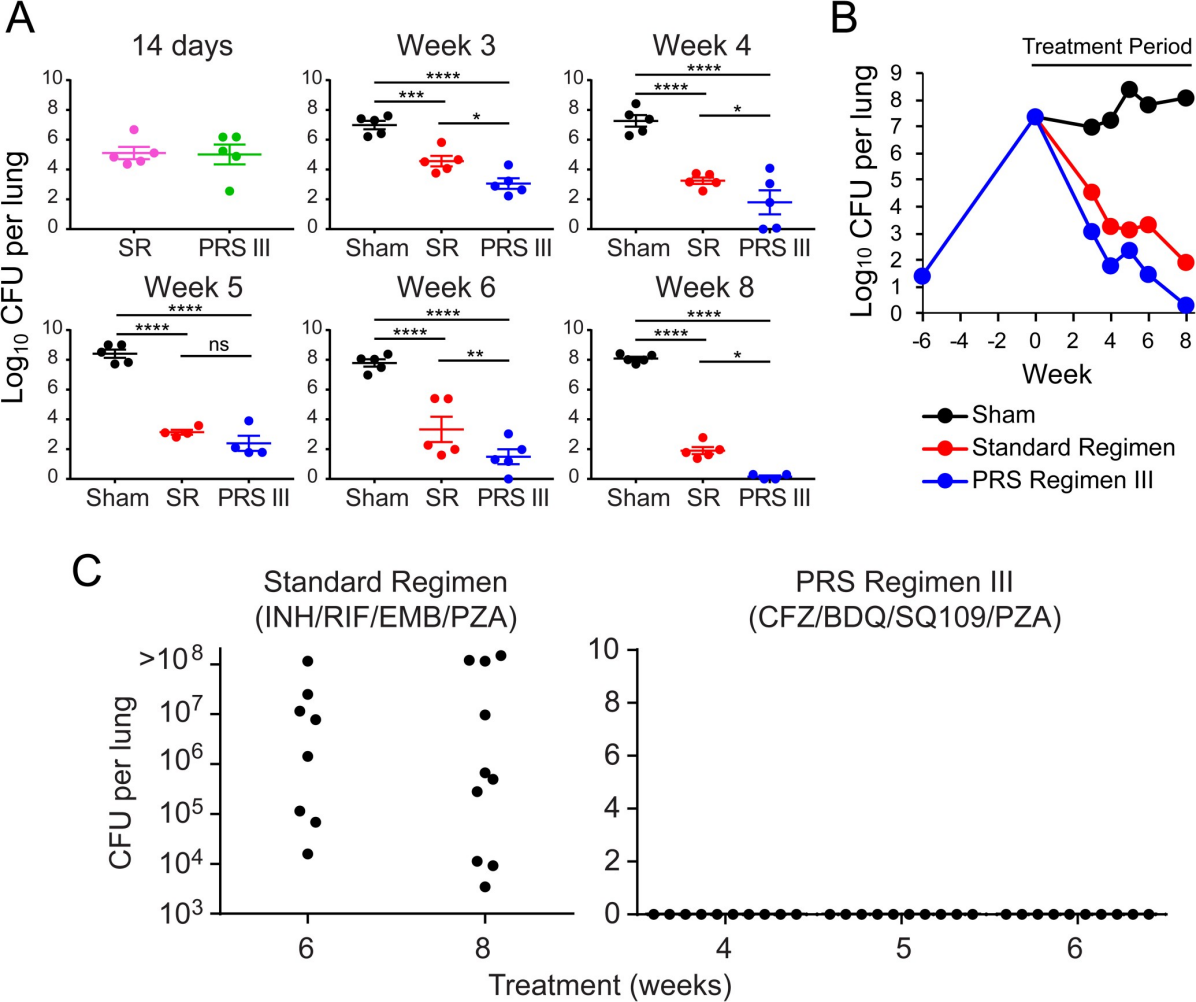
Treatment efficacy and Time to Relapse-free Cure study in C3HeB/FeJ mice. (A) Lung burden of *M*. *tuberculosis* after treatment daily for 14 days or 5 days per week for 3, 4, 5, 6 and 8 weeks in sham-treated mice or mice treated with the Standard Regimen (SR) or PRS Regimen III (PRS III), d, daily. Two-way ANOVA with Tukey’s multiple comparison test was used in statistical analyses. **** *p* < 0.0001, *** *p* < 0.001, ** *p* < 0.01, * *p* < 0.05, ns, not significant (B) Lung burden of *M*. *tuberculosis* over the course of infection and treatment. d, daily (C) Total number of *M*. *tuberculosis* in the lung of each mouse was determined three months after treatment cessation. Relapse is defined as 1 or more CFU per lung. For the Standard Regimen group at 8 weeks, and PRS Regimen III groups at 4 and 6 weeks, n = 10 mice/group. For Standard Regimen group at 6 week, n = 8 mice/group, and the PRS Regimen III group at 5 weeks, n = 9 mice/group. (*p* <0.0001, PRS Regimen III versus Standard Regimen, log rank test).

**Fig 5:**
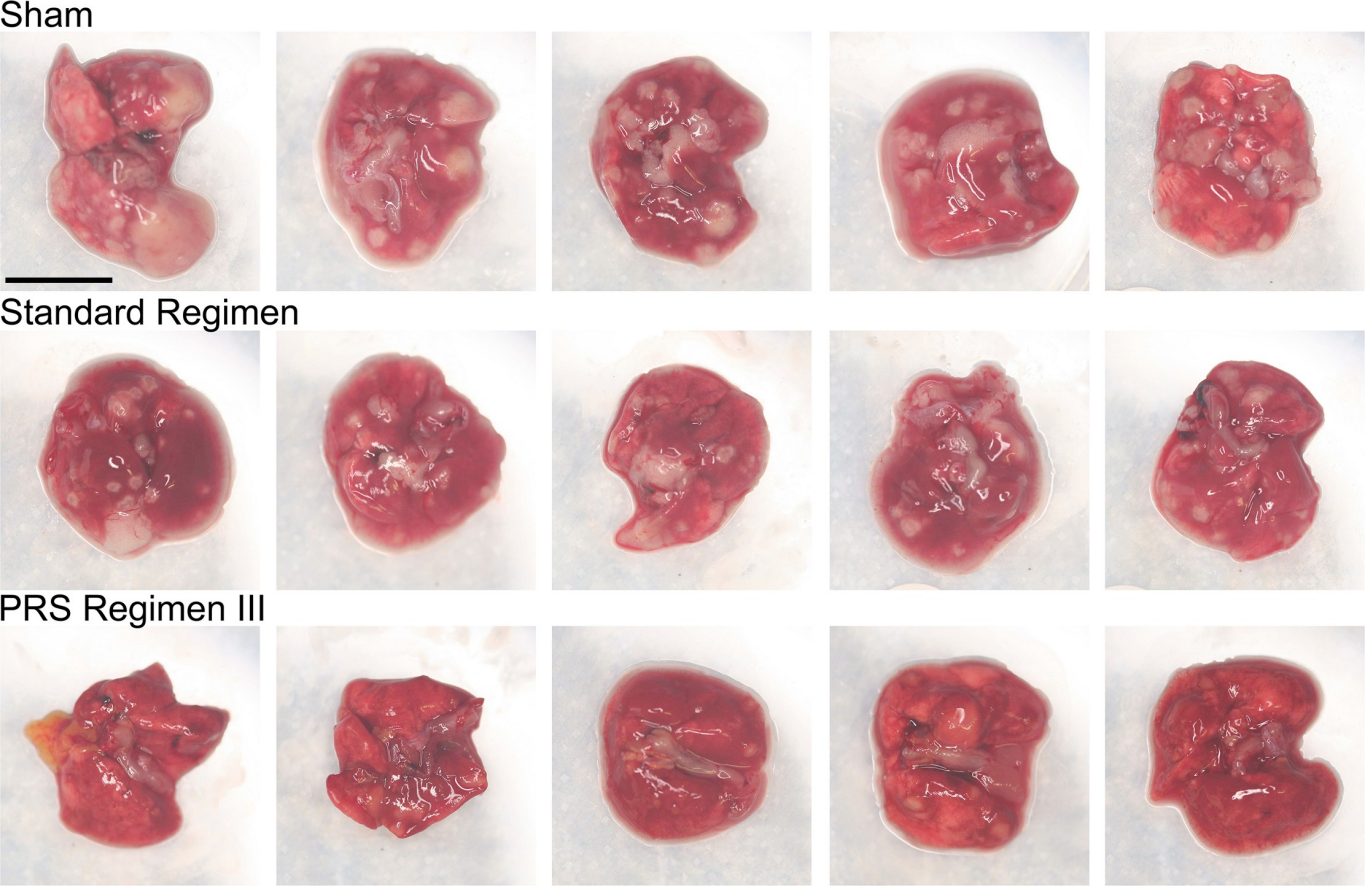
Lung gross pathology in C3HeB/FeJ mice upon completion of 4 weeks of treatment. Mice (n = 5 per group) were infected with *M*. *tuberculosis* by aerosol and starting six weeks later, they were either sham-treated or treated with the Standard Regimen (INH/RIF/EMB/PZA at 25/10/100/150 mg/kg), PRS Regimen II (CFZ/BDQ/EMB/PZA at 25/30/100/450 mg/kg) or PRS Regimen III (CFZ/BDQ/SQ109/PZA at 25/30/25/450 mg/kg) 5 days per week for 4 weeks. Their lungs and surface granulomas were photographed for inspection. Note that granulomas of the sham treated mice are much larger in the C3HeB/FeJ mice than the BALB/c mice in Fig 3. Scale bar (upper left panel), 1 cm.

C3HeB/FeJ mice develop necrotic lung lesions, similar to those found in human lungs with TB [14,18], prompting us to assess the efficacy of PRS Regimen III versus the Standard Regimen in this mouse model. We infected the mice and waited 6 weeks until the lung burden had reached a high level of 7.4 log_10_ CFU, 1.6 logs higher than that in the long-term study in the BALB/c mouse model, before starting the treatment (S2C and S5A Tables). By this time point, very large granulomas were evident in the mouse lungs. In an EBA_14_ study, in which mice were treated daily for 14 days, there was no significant difference in the rate of CFU reduction between mice treated with the Standard Regimen versus PRS Regimen III (Fig 4A). However, in a long-term efficacy and relapse study, in which mice were treated 5 days per week, PRS Regimen III led to a greater reduction than the Standard Regimen in the lung burden of *M*. *tuberculosis* at 3 weeks and thereafter (Fig 4A and 4B and S5A Table). We assessed relapse in the mice after completion of treatment with PRS Regimen III for 4, 5 or 6 weeks and with the Standard Regimen for 6 or 8 weeks (Fig 4C and S5B Table). Treatment with PRS Regimen III achieved relapse-free cure at 4 weeks; in contrast, 100% of mice treated with the Standard Regimen relapsed at 6 weeks and 8 weeks (*p* <0.0001, PRS Regimen III versus Standard Regimen, log rank test). At necropsy, mice treated with PRS Regimen III had fewer lesions on the surface of their lungs than mice treated with the Standard Regimen at all time points. This was true both during the course of treatment and during the period of relapse assessment (Fig 5 and S3 and S4 Figs).

## Discussion

In our initial application of the PRS platform to identify improved drug regimens for treating TB, we identified, from a pool of 14 different TB drugs, many regimens among the 1365 possible 3- or 4-drug regimens that are more efficacious than the Standard Regimen in inhibiting *M*. *tuberculosis* in their host cells—human macrophages [7]. Subsequently, in a proof-of-concept study, we showed that two such regimens were much more efficacious than the Standard Regimen in curing TB in the mouse model of pulmonary TB; one such regimen, PRS Regimen II, reduced the time to relapse-free cure by 75% compared with the Standard Regimen [5]. However, as this regimen included two first-line drugs, EMB and PZA, it was not suitable for treating drug-resistant TB, for which three non-first-line drugs are needed to prevent the emergence of resistance among the enormous population of TB bacilli typically present in the lungs in human infection. Hence, in the present study, with the aim of identifying a regimen that was not only rapidly effective but also suitable for treating drug-resistant TB, we substituted the investigational drug SQ109 for EMB in PRS Regimen II, as the PRS platform indicated that these drugs are interchangeable with respect to the efficacy of regimens containing them. We show that the resultant regimen, PRS Regimen III, is at least as efficacious as PRS Regimen II in rapidly curing TB in two different mouse models of TB, BALB/c and C3HeB/FeJ. PZA is the only first-line drug included in the PRS Regimen III. The incidence of PZA-resistance in MDRTB isolates varies from country-to-country, but is typically about 40% [19–23]. Therefore, approximately 60% of cases of MDRTB would have no resistance to any of the drugs in our regimen.

Because the routinely used BALB/c mouse model does not develop the caseous necrotic lesions of pulmonary TB that are seen in human TB, there has been concern that preclinical drug studies in BALB/c mouse may not be as predictive of findings in humans as those in C3HeB/FeJ mice, which do develop such caseous necrotic lesions, particularly if the antibiotics being tested have lower activity in or lower penetration into regions of caseous necrosis. Nevertheless, we observed that PRS Regimens II and III lead to relapse-free cure in C3HeB/FeJ mice just as rapidly as in BALB/c mice. Similarly, Li et al. reported that the Standard Regimen as well as other drug combinations reduced lung CFU in BALB/c and C3HeB/FeJ mice to a similar extent [24]. Hence, while individual drugs have shown disparate activity in the two mouse models [25–28], thus far, combination therapies have shown similar activity. Combination drug regimens that target multiple pathways and act synergistically may effectively treat both intracellular and extracellular *M*. *tuberculosis* and erase differences between these two mouse strains that are seen with drug monotherapy.

PRS Regimens III differs from PRS Regimen II by replacing EMB with SQ109. SQ109 has several advantages over EMB: 1) higher potency [29], 2) extremely low spontaneous mutation rate [30], 3) active against MDRTB and even XDRTB [31], and 4) while EMB may cause significant side effects, including optic neuropathy, SQ109 has been well tolerated in Phase I and Phase II clinical trials [31]. A study led by Nikonenko and co-workers showed that combining the three first-line drugs INH, RIF and PZA with SQ109 instead of EMB was more effective and resulted in a significantly greater reduction in lung burden of *M*. *tuberculosis* over 8 weeks treatment in a mouse model of TB [32]. Sacksteder et al. reported that addition of SQ109 to BDQ and PZA, a two-drug combination, cut the time required to obtain a negative culture of *M*. *tuberculosis* in the lung by a month from 12 weeks to 8 weeks [31]. Ultimately, SQ109’s inclusion in PRS Regimen III may prove to be important in preventing development of drug resistance, just as EMB serves this purpose in the Standard Regimen.

Because CFZ was held constant in the dose efficacy response mapping study, its contribution to efficacy cannot be ascertained in that study. However, in earlier in vitro studies [7] of the efficacy of various drug combinations on the viability of intramacrophge *M*. *tuberculosis*, combinations containing CFZ were consistently among the most potent. For example, the top six 4-drug combinations with a projected inhibition greater than 90% all included CFZ (Inhibition 93%-98% vs. 85% for the Standard Regimen) [7]. An analysis of data for just the combination CFZ, BDQ and PZA in an iteration in which the Standard Regimen had a projected inhibition of 53% showed that dropping CFZ from the combination of CFZ, PZA, and BDQ markedly reduced the level of inhibition from 69% to 53%.

Poor adherence to a treatment regimen increases the risk of treatment failure. Multiple factors, including the length of treatment, impact patient adherence to TB treatment. It was reported by Combs and colleagues from the clinical trial comparing a 6-month regimen comprised of INH and RIF supplemented with PZA in the first 2 months with a 9-month standard regimen of INH and RIF that not only was the 6-month “short course treatment” more effective but also that significantly more patients adhered to the treatment, which they attributed to the shorter duration of therapy [1]. This observation supports the concept that a highly effective regimen which sterilizes the lung after a short course of treatment has potential for a higher rate of treatment success than a less effective regimen which requires a lengthy course of treatment. As many toxicities depend on both dose and duration of treatment, the ultra-short course treatment may also reduce overall risk of treatment-related toxicity. As noted in our Supporting Information (S1 Text), the drug doses that we are using in mice in PRS III are compatible with what can be achieved readily in human dosing.

An intriguing finding in our studies is that, while the treatment time required to attain lung sterilization and the treatment time required to attain relapse-free cure are linked, they are not the same. We have observed in our studies of PRS Regimens II and III that if the lung CFU is below 10 when drug dosing ceases, the mice often will be free of relapse three months later. At least two factors may contribute to this phenomenon: 1) the mouse immune system may be able to control and eliminate low numbers of organisms remaining after treatment completion and, perhaps more importantly, 2) the drugs in PRS Regimens II and III have very long half-lives and continue to kill the remaining *M*. *tuberculosis* long after drug dosing ceases. For example, in humans the half-life of BDQ is 5.5 months [33] and the half-life of CFZ is at least 70 days [34]. Their half-lives in mice are shorter, e.g. BDQ has a plasma half-life of 50–60 hours after a single dose [35] and CFZ has a half-life of 4-weeks in the lungs after 4-weeks of repeated dosing [36] but are still long enough to contribute to continued efficacy after the cessation of dosing. Therefore, even when drug dosing is stopped at 3 weeks, and at this point there are still a few residual CFU in mouse organs, therapeutic levels of BDQ and CFZ may persist for a sufficient period of time to achieve complete sterilization and relapse-free cure. These two mechanisms of control and eradication–immunity and the extraordinarily long drug half-lives of BDQ and CFZ–probably work in concert to achieve sterilization and relapse-free cure despite a small number of residual organisms at the time of dosing cessation. In part because of the long half-lives of these drugs, we suspect that appropriate drug regimens containing them will enable a shorter treatment course of TB in humans.

In conclusion, our study shows that PRS Regimen III has early bactericidal activity ~3-fold greater than that of the Standard Regimen used to treat TB and markedly shortens the time required both to sterilize the lung and to achieve relapse-free cure in rigorous mouse models of pulmonary TB. Studies in the mouse model of TB have been broadly predictive of treatment efficacy in human clinical trials. However, whether the dramatic shortening of treatment that we observe in our two mouse models using PRS Regimen III also applies to humans awaits testing in clinical trials. If successful, PRS Regimen III, involving treatment with CFZ, BDQ, PZA, and SQ109, has the potential to provide an ultra-short course treatment effective both for drug-sensitive and most, if not all, cases of drug-resistant TB.

## Supporting information

S1 Text Drug dosing levels: Comparison of mouse and human dosing of PRS Regimen III. (PDF)

S1 Table BALB/c mouse lung burden of *M*. *tuberculosis* in PRS Regimen III optimal dose finding study. (PDF)

S2 Table Scheme of treatment efficacy and Time to Relapse-free Cure studies in BALB/c and C3HeB/FeJ models of pulmonary tuberculosis. (A) Short-term efficacy and relapse study in BALB/c mice, (B) Long-term efficacy and relapse study in BALB/c mice, (C) Treatment efficacy and relapse study in C3HeB/FeJ mice.
(PDF)

S3 Table BALB/c mouse lung burden of *M*. *tuberculosis* in short-term efficacy and relapse study. (A) Short-term efficacy, (B) Relapse: Total lung CFU 3 months after treatment with the Standard Regimen or Enhanced Standard Regimen for the period indicated, (C) Relapse: Total lung CFU 3 months after treatment with PRS Regimen II or III for the period indicated.
(PDF)

S4 Table BALB/c mouse lung burden of *M*. *tuberculosis* in long-term efficacy and relapse study. (A) Time to Lung Sterilization study, (B) Time to Relapse-free Cure study: Standard Regimen, (C) Time to Relapse-free Cure study: PRS Regimen II, (D) Time to Relapse-free Cure Study: PRS Regimen III.
(PDF)

S5 Table C3HeB/FeJ mouse lung burden of *M*. *tuberculosis* in treatment efficacy and relapse study. (A) Efficacy, (B) Relapse: Total lung CFU 3 months after treatment with the Standard Regimen or PRS Regimen III for the period indicated.
(PDF)

S1 Fig Short-term efficacy and relapse study. (A) *M*. *tuberculosis* burdens in the lung were determined over the course of infection and treatment period. (B) Lung burden of *M*. *tuberculosis* after treatment 5 days per week by oral gavage for 2, 3, and 4 weeks in sham-treated mice or mice treated with the Standard Regimen (SR), Enhanced Standard Regimen (ESR), PRS Regimen II (PRS II) or PRS Regimen III (PRS III). For mice with zero CFU in the lungs, a CFU count of 1 was assigned for graphing purposes. Two-way ANOVA with Tukey’s multiple comparison test was used in statistical analyses. **** *p* < 0.0001, * *p* < 0.05, ns, not significant (C) Number of *M*. *tuberculosis* organisms in the lung of each mouse 3 months after treatment cessation.
(PDF)

S2 Fig Lung gross pathology in BALB/c mice upon completion of 3, 5, and 6 weeks of treatment. Mice were infected with *M*. *tuberculosis* by aerosol and starting two weeks later were sham-treated or treated with the Standard Regimen (INH/RIF/EMB/PZA at 25/10/100/150 mg/kg), PRS Regimen II (CFZ/BDQ/EMB/PZA at 25/30/100/450 mg/kg) or PRS Regimen III (CFZ/BDQ/SQ109/PZA at 25/30/25/450 mg/kg) 5 days per week for (A) 3 weeks, (B) 5 weeks or (C) 6 weeks. The mice were then euthanized and their lungs and surface granulomas photographed. Scale bar (upper left panel), 1 cm.
(PDF)

S3 Fig Lung gross pathology in C3HeB/FeJ mice upon completion of 3, 5, and 6, and 8 weeks of treatment. Mice were infected with *M*. *tuberculosis* by aerosol and starting six weeks later were sham-treated or treated with the Standard Regimen (INH/RIF/EMB/PZA at 25/10/100/150 mg/kg) or PRS Regimen III (CFZ/BDQ/SQ109/PZA at 25/30/25/450 mg/kg) 5 days per week for (A) 3 weeks, (B) 5 weeks, (C) 6 weeks, and (D) 8 weeks. The mice were then euthanized and their lungs and surface granulomas photographed. Scale bar (upper left panel), 1 cm.
(PDF)

S4 Fig Lung gross pathology in C3HeB/FeJ mice 3 months after cessation of treatment. Photographs show the lungs and surface granulomas from mice 3 months after completion of treatment with the Standard Regimen (INH/RIF/EMB/PZA at 25/10/100/150 mg/kg) or PRS Regimen III (CFZ/BDQ/SQ109/PZA at 25/30/25/450 mg/kg) for (A) 4 weeks, (B) 5 weeks or (C) 6 weeks. Scale bar (upper left panel), 1 cm.
(PDF)

S1 Code PRS Regimen III MATLAB code. (PDF)
