# Supplementary material for: Ultra-rapid near universal TB drug regimen identified via parabolic response surface platform cures mice of both conventional and high susceptibility
Source: PLoS One. 2018 Nov 14;13(11):e0207469. doi: 10.1371/journal.pone.0207469 (PMC6235396; doi:10.1371/journal.pone.0207469)
Supplement: S1 Text — (PDF) [file pone.0207469.s001.pdf]

S1 Text

Drug Dosing Levels: Comparison of Mouse and Human Dosing of PRS Regimen III

As discussed below, the dosing used in PRS Regimen III is achievable in human dosing.

PZA: The optimized dose of PZA in PRS Regimens II and III is 450 mg/kg, 3 times the 150 mg/kg dose commonly tested in mice. According to FDA body surface area based dose-conversion guidance, a dose of 450 mg/kg in the mouse corresponds to 36.6 mg/kg in a 60 kg human ('FDA Guidance for Industry: Estimating the Maximum Safe Starting Dose in Initial Clinical Trials for Therapeutics in Adult Healthy Volunteers', available at http://www.fda.gov/downloads/Drugs/.../Guidances/UCM078932.pdf). This dose is only slightly higher than the clinically recommended dose, just above the upper end of the 15-30 mg/kg dosing range recommended by CDC – ATS guidelines, and, for anyone under 55 kg, within the 2 gram maximum recommended dose. Moreover, according to the Handbook of anti-TB Agents [1], a human dose of 27 mg/kg gives an AUC of 502 mg hr/L, whereas a mouse dose of 150 mg/kg gives an AUC of only 303.8 mg h/L. Thus, a 150 mg/kg dose in the mouse clearly falls substantially short of bioequivalence to the standard human dose.

With respect to toxicity of PZA in humans, higher doses of PZA have been given to humans without a dose-dependent increase in hepatotoxicity. Indeed, Pasipanodya and Gumbo performed a detailed meta-analysis of human clinical trials, including clinical trials with doses as high as 50 mg/kg and 60 mg/kg and concluded, as their title states, that "High-Dose Pyrazinamide Is Not More Hepatotoxic than the Low Doses Currently Used" [2]. While there are dose-dependent increases in arthralgias and hyperuricemia, these are manageable side effects for which the toxicity/benefit ratio is likely to be acceptable if it yields a more effective and faster cure. It is also important to note that in the Standard Regimen, PZA is given in combination with two other hepatotoxic drugs (INH and RIF), but that is not the case with our PRS regimens. BDQ has been associated with mild elevations of serum levels of hepatic transaminases, but these have almost always been asymptomatic and self-limited in duration, and it is unclear as to which drug in the regimen was responsible. On the other hand, the hepatotoxicity of INH and RIF is very well established.

While the 450 mg pyrazinamide dose may be ideal, the drug is still highly effective at lower doses. As is clear from our dose response mapping (Fig. 1), the dose response surface for PZA has a very gradual slope and the regimen remains highly effective even at much lower doses of PZA. Thus, if the higher dose of PZA is not tolerated, our data indicate that the dose can be reduced without greatly decreasing efficacy. The flat dose-response surface for PZA suggests the possibility that the regimen would be highly effective even without PZA (in which case the regimen would also be expected to be about as effective against PZA-resistant as against PZA-sensitive strains of *M. tuberculosis*); however, whether this is so remains to be investigated.

BDQ: With respect to BDQ, current standard clinical BDQ dosing is 400 mg once daily for 2 weeks, followed by 200 mg three times/week thereafter (which would average out to 120 mg/day if given 5 times per week, as in our Monday-Friday dosing). Following the FDA guidance for mouse – to – human dose conversion, our mouse dose of 30 mg/kg converts to a human dose of 2.4 mg/kg, i.e., a dose of 144 mg for a 60 kg human. The correspondence of this dosing level is confirmed by AUC measurements after repeated dosing in mice and humans. The AUC in mice for BDQ after 6 weeks of 5 day/week dosing at 15, 30, and 60 mg/kg are approximately 12, 21, and 48 μg h/ml, respectively [3]. In humans, a BDQ daily oral dose of 400 mg administered for a week resulted in an AUC of 64.75 μg h/ml, and studies have shown that for both humans and mice, the AUC is linear with dose. Therefore, our dose of 30 mg/kg in mice corresponds to an AUC of 21 μg h/ml, which in turn corresponds to a human dose of 130 mg/kg daily (21/64.75 x 150mg/kg) i.e., in good agreement with the FDA body-surface area based guidance for dose conversion. Our FDA guidance based dose regimen would use a constant level of 144 mg, which is comparable to the current standard dosing, and therefore readily achievable.

SQ109: With respect to SQ109, based on the FDA dose conversion guidance, our mouse dose of 25 mg/kg corresponds to a human dose of 2 mg/kg, i.e. a dose of 120 mg SQ109 for a 60 kg human. The doses of SQ109 that are being used in clinical trials are 75 mg, 150 mg, and 300 mg once daily [4]. Therefore, our mouse dose for SQ109 falls in the middle of the human dosing in use in clinical trials.

CFZ: With respect to CFZ, our mouse dose of 25 mg/kg converts to a human dose of 2 mg/kg, or 120 mg for a 60 kg human based on the FDA dose conversion guidance. The dose of CFZ recommended for lepromatous leprosy complicated by erythema nodosum is 100 mg to 200 mg

daily for up to 3 months, with dose tapering down to 100 mg after that 3-month period [5]. Thus the CFZ dosing in our regimen, which would convert to a human dose of 120 mg for less than 6 weeks, is readily achievable and less than what is often used clinically for leprosy. Moreover, because the duration of treatment is considerably shorter than that required for treatment of leprosy, we anticipate much less intolerance due to gastrointestinal symptoms and skin discoloration.

References

1. Global Alliance for TB Drug Development. Handbook of anti-tuberculosis agents. Tuberculosis (Edinb) 2008;88: 85-86.
2. Pasipanodya JG, Gumbo T. Clinical and toxicodynamic evidence that high-dose pyrazinamide is not more hepatotoxic than the low doses currently used. Antimicrob Agents Chemother. 2010;54: 2847-2854.
3. Rouan MC, Lounis N, Gevers T, Dillen L, Gilissen R, Raoof A, et al. Pharmacokinetics and pharmacodynamics of TMC207 and its N-desmethyl metabolite in a murine model of tuberculosis. Antimicrob Agents Chemother. 2012;56: 1444-1451.
4. Heinrich N, Dawson R, du Bois J, Narunsky K, Horwith G, Phipps AJ, et al. Early phase evaluation of SQ109 alone and in combination with rifampicin in pulmonary TB patients. J Antimicrob Chemother. 2015;70: 1558-1566.
5. Novartis. Lamprene (clofazimine) prescribing information. 2002. Available from: https://wwwaccessdatafdagov/drugsatfda_docs/label/2016/019500s013lblpdf. East Hanover, NJ
