# Supplementary material for: Ultra-rapid near universal TB drug regimen identified via parabolic response surface platform cures mice of both conventional and high susceptibility
Source: PLoS One. 2018 Nov 14;13(11):e0207469. doi: 10.1371/journal.pone.0207469 (PMC6235396; doi:10.1371/journal.pone.0207469)
Supplement: S1 Table — (PDF) [file pone.0207469.s002.pdf]

S1 Table. BALB/c mouse lung burden of *M. tuberculosis* in PRS Regimen III optimal dose finding study.

Treatment	Concentration (mg/kg)	Log_{10} CFU (Mean ± SEM)*		
		Day 1	Day 14	Day 35
Sham		2.23 ± 0.09	6.00 ± 0.04	7.17 ± 0.12
Standard Regimen				
RIF/EMB/INH/PZA	10/100/25/150			4.38 ± 0.07
PRS Regimen II				
CFZ/EMB/BDQ/PZA	25/100/30/450			0.57 ± 0.15
PRS Regimen III				
CFZ/SQ109/BDQ/PZA	25/2.78/5.6/450			1.39 ± 0.14
	25/2.78/50/50			0.75 ± 0.08
	25/25/5.6/50			3.21 ± 0.10
	25/25/50/450			0.23 ± 0.16†
	25/25/50/50			0.61 ± 0.17
	25/25/5.6/450			1.63 ± 0.08
	25/2.78/50/450			0.33 ± 0.14†
	25/25/16.7/150			1.05 ± 0.16
	25/8.25/50/150			0.65 ± 0.10
	25/8.25/16.7/450			0.54 ± 0.15
	25/16.75/50/450			0.36 ± 0.15
	25/8.25/50/450			0.34 ± 0.14†

*BALB/c mice (n = 5 per group) were infected with *M. tuberculosis* by aerosol on Day 0. Two mice were euthanized on Day 1 to determine the number of bacteria delivered to the lungs. Three mice were euthanized on Day 14 to determine the bacterial level in the lung just prior to treatment. Starting on Day 14, mice were sham treated or treated with the Standard Regimen, PRS Regimen II or various concentrations of PRS Regimen III 5 days per week (Monday – Friday) for 3 weeks. Mice were euthanized 3 days after the last treatment (Day 35), and the entire lung was homogenized and plated to determine log_{10} CFU in the lung.

†One mouse in each of the indicated groups had zero CFU in their entire lung for which a lung CFU count of 1 was assigned for the log_{10} CFU calculation.
