# Supplementary material for: Ultra-rapid near universal TB drug regimen identified via parabolic response surface platform cures mice of both conventional and high susceptibility
Source: PLoS One. 2018 Nov 14;13(11):e0207469. doi: 10.1371/journal.pone.0207469 (PMC6235396; doi:10.1371/journal.pone.0207469)
Supplement: S2 Table — (A) Short-term efficacy and relapse study in BALB/c mice, (B) Long-term efficacy and relapse study in BALB/c mice, (C) Treatment efficacy and relapse study in C3HeB/FeJ mice. (PDF) [file pone.0207469.s003.pdf]

S2 Table. Scheme of treatment efficacy and relapse studies in BALB/c and C3HeB/FeJ models of pulmonary tuberculosis.

(A) Short-term efficacy and relapse study in BALB/c mice

**Treatment**†	**Mice**	**Week (Number of Mice Euthanized per Group*)**				
		**-2**	**0**	**2**	**3**	**4**
Untreated	8	3	5			
Sham	15			5	5	5
Standard Regimen	20			5	5	5
						**5**
Enhanced Standard Regimen	20			5	5	5
						**5**
PRS Regimen II	30			5	5	5
				**5**	**5**	**5**
PRS Regimen III	30			5	5	5
				**5**	**5**	**5**

(B) Long-term efficacy and relapse study in BALB/c mice

**Treatment***	**Mice**	**Week (Number of Mice Euthanized per Group†)**											
		**-2**	**0**	**2**	**3**	**4**	**5**	**6**	**8**	**12**	**16**	**20**	**22**
Untreated	8	3	5										
Sham	40				5	5	5	5	5	5	5	5	
Standard Regimen‡	94				5	5	5	5	5	5	5	5	
									**10**	**10**	**10**	**14**	**10**
PRS Regimen II	60				5	5	5	5					
					**10**	**10**	**10**	**10**					
PRS Regimen III	57				5	5	5	5					
					**9**	**8**	**10**	**10**					

(C) Treatment efficacy and relapse study in C3HeB/FeJ mice

**Treatment***	**Mice**	**Week (Number of Mice Euthanized per Group*)**							
		**-6**	**0**	**2**	**3**	**4**	**5**	**6**	**8**
Untreated	8	3	5						
Sham	25				5	5	5	5	5
Standard Regimen	43				5	5	5	5	5
								**8**	**10**
PRS Regimen III	54				5	5	5	5	5
						**10**	**9**	**10**	
Standard Regimen (daily)	5			5					
PRS Regimen III (daily)	5			5					

*Starting 2 weeks (BALB/c mice) or 6 weeks (C3HeB/FeJ mice) after aerosol infection, mice were treated by oral gavage daily for 5 days per week with the treatment regimen indicated.

†Non-bolded numbers indicate the number of mice per group euthanized 3 days after completion of treatment for the time indicated to determine their lung burden of *M. tuberculosis*. Bold numbers indicate the number of mice per group euthanized 3 months after completion of treatment for the time indicated to determine their lung burden of *M. tuberculosis*.

‡For the long-term study, mice treated with the Standard Regimen were administered RIF/EMB/INH/PZA by gavage for up to 8 weeks, and thereafter administered RIF and INH only.
