# Supplementary material for: Ultra-rapid near universal TB drug regimen identified via parabolic response surface platform cures mice of both conventional and high susceptibility
Source: PLoS One. 2018 Nov 14;13(11):e0207469. doi: 10.1371/journal.pone.0207469 (PMC6235396; doi:10.1371/journal.pone.0207469)
Supplement: S3 Table — (A) Short-term efficacy, (B) Relapse: Total lung CFU 3 months after treatment with the Standard Regimen or Enhanced Standard Regimen for the period indicated, (C) Relapse: Total lung CFU 3 months after treatment with PRS Regimen II or III for the period indicated. (PDF) [file pone.0207469.s004.pdf]

S3 Table. BALB/c mouse lung burden of *M. tuberculosis* in short-term efficacy and relapse study.

(A) Short-term Efficacy

Treatment Week	Sham	Standard Regimen	Enhanced Standard Regimen	PRS Regimen II	PRS Regimen III
-2	2.54 ± 0.06				
0	6.13 ± 0.03				
2	6.61 ± 0.13	4.15 ± 0.07	3.93 ± 0.05	2.56 ± 0.09	2.60 ± 0.15
3	6.81 ± 0.29	3.52 ± 0.06	2.96 ± 0.11	0.41 ± 0.17	0.91 ± 0.32
4	6.69 ± 0.17	3.08 ± 0.06	2.47 ± 0.07	0.10 ± 0.10	0.00 ± 0.00§

(B) Relapse: Total lung CFU 3 months after treatment with the Standard Regimen or Enhanced Standard Regimen for the period indicated

	Standard Regimen			Enhanced Standard Regimen		
**Mouse**	2 weeks	3 weeks	4 weeks	2 weeks	3 weeks	4 weeks
1	n.d.	n.d.	15,470	n.d.	n.d.	53,705
2	n.d.	n.d.	34,970	n.d.	n.d.	38,875
3	n.d.	n.d.	53,190	n.d.	n.d.	98,750
4	n.d.	n.d.	35,760	n.d.	n.d.	9,450
5	n.d.	n.d.	29,160	n.d.	n.d.	1,190,000

(C) Relapse: Total lung CFU 3 months after treatment with PRS Regimen II or III for the period indicated

	PRS Regimen II			PRS Regimen III		
**Mouse**	2 weeks	3 weeks	4 weeks	2 weeks	3 weeks	4 weeks
1	1,236	0	1	3,120	1,216	8,333
2	499	2,593	4	12,000	0	5
3	10,000	0	1	1,080	0	1
4	5,347	1,760	2	942	0	0
5	4,050	1,095	32	6,460	984	1

Starting two weeks after aerosol infection, BALB/c mice (n = 5 per group) were treated 5 days per week (Monday-Friday) for 2, 3, or 4 weeks. (A) For the short-term efficacy study, lung log_{10} CFU were determined three days after the last treatment. At the end of 4 weeks treatment, 4 out

of 5 mice treated with PRS Regimen II and all 5 mice treated with PRS Regimen III had zero CFU in the entire lung; these mice were assigned a lung CFU count of 1 for the $\log_{10}$ CFU calculation. Data shown are mean ± SEM. §All mice in this group had zero CFU in the entire lung. (B, C) For the relapse study, mice were held for 3 months after the last treatment dose and then euthanized for assay of lung CFU. Data are total lung CFU for each animal. n.d., not done
