# Supplementary material for: Ultra-rapid near universal TB drug regimen identified via parabolic response surface platform cures mice of both conventional and high susceptibility
Source: PLoS One. 2018 Nov 14;13(11):e0207469. doi: 10.1371/journal.pone.0207469 (PMC6235396; doi:10.1371/journal.pone.0207469)
Supplement: S4 Table — (A) Time to Lung Sterilization study, (B) Time to Relapse-free Cure study: Standard Regimen, (C) Time to Relapse-free Cure study: PRS Regimen II, (D) Time to Relapse-free Cure Study: PRS Regimen III. (PDF) [file pone.0207469.s005.pdf]

S4 Table. BALB/c mouse lung burden of *M. tuberculosis* in Time to Lung Sterilization study and Time to Relapse-free Cure study.

(A) Time to Lung Sterilization Study*

Treatment† Week	Sham	Standard Regimen	PRS Regimen II	PRS Regimen III
	Log_{10} CFU	Log_{10} CFU [Total CFU]	Log_{10} CFU [Total CFU]	Log_{10} CFU [Total CFU]
-2	2.48 ± 0.01			
0	5.76 ± 0.02			
3	6.82 ± 0.08	3.54 ± 0.07	1.46 ± 0.14	1.13 ± 0.15
4	6.77 ± 0.09	3.71 ± 0.09	[6.00 ± 3.36]	[3.00 ± 0.40]
5	5.72 ± 0.43	3.56 ± 0.08	[0.20 ± 0.20]	[0.00 ± 0.00]
6	6.60 ± 0.04	3.07 ± 0.08	[0.00 ± 0.00]	[0.00 ± 0.00]
8	6.55 ± 0.06	2.05 ± 0.08		
12	6.47 ± 0.10	[2.20 ± 0.58]		
16	6.64 ± 0.14	[0.00 ± 0.00]		
20	6.75 ± 0.12	[0.00 ± 0.00]		

(B) Time to Relapse-free Cure Study: Standard Regimen*‡

	**Total lung CFU 3 months after treatment for time indicated**				
**Mouse**	8 weeks	12 weeks	16 weeks	20 weeks	22 weeks
1	6,175	1,628	0	0	0
2	7,857	1	722	0	0
3	2,288	0	0	0	0
4	26,500	2	0	0	0
5	2,123	3,960	0	0	0
6	31,320	1	0	0	0
7	17,928	0	0	0	0
8	9,650	5,021	0	0	0
9	4,108	6,622	0	0	0
10	2,500	4,930	0	0	0
11				0	
12				0	
13				0	
14				0	

(C) Time to Relapse-free Cure Study: PRS Regimen II*‡

Mouse	Total lung CFU 3 months after treatment for time indicated			
	3 weeks	4 weeks	5 weeks	6 weeks
1	2,550	0	0	0
2	0	0	0	0
3	608	0	0	0
4	0	0	0	0
5	1,462	0	0	0
6	0	0	0	0
7	0	0	0	0
8	0	0	0	0
9	0	0	0	0
10	0	0	0	0

(D) Time to Relapse-free Cure Study: PRS Regimen III*‡

Mouse	Total lung CFU 3 months after treatment for time indicated			
	3 weeks	4 weeks	5 weeks	6 weeks
1	0	0	0	0
2	0	0	0	0
3	0	0	0	0
4	924	0	0	0
5	0	0	0	0
6	0	0	0	0
7	4	0	0	0
8	5,500	0	0	0
9	1,156		0	0
10			0	0

*Starting two weeks after aerosol infection, mice were treated 5 days per week (Monday-Friday).

†For the Time to Lung Sterilization study, lung CFU was determined three days after the last treatment. At the end of 5 weeks treatment, four out of five mice treated with PRS Regimen II and all five mice treated with PRS Regimen III had zero CFU in the lung. Data shown are mean ± SEM of CFU counts in the entire organ, either as $\log_{10}$ CFU, or if in brackets, as total CFU counts.

‡For the Time to Relapse-free Cure study, mice were held for 3 months after the last treatment dose and then euthanized for assay of lung CFU. Data are total lung CFU for each animal.
