# Supplementary material for: Ultra-rapid near universal TB drug regimen identified via parabolic response surface platform cures mice of both conventional and high susceptibility
Source: PLoS One. 2018 Nov 14;13(11):e0207469. doi: 10.1371/journal.pone.0207469 (PMC6235396; doi:10.1371/journal.pone.0207469)
Supplement: S5 Table — (A) Efficacy, (B) Relapse: Total lung CFU 3 months after treatment with the Standard Regimen or PRS Regimen III for the period indicated. (PDF) [file pone.0207469.s006.pdf]

S5 Table. C3HeB/FeJ mouse lung burden of *M. tuberculosis* in treatment efficacy and relapse study.

(A) Efficacy*†

**Treatment Week**	**Sham**	**Standard Regimen**	**PRS Regimen III**
0	7.37 ± 0.28		
2	n.d.	5.11 ± 0.41	5.01 ± 0.07
3	6.99 ± 0.28	4.57 ± 0.35	3.06 ± 0.35
4	7.27 ± 0.38	3.25 ± 0.21	1.80 ± 0.81
5	8.41 ± 0.28	3.14 ± 0.16§	2.39 ± 0.51§
6	7.79 ± 0.25	3.33 ± 0.85	1.50 ± 0.50
8	8.09 ± 0.12	1.91 ± 0.24	0.30 ± 0.09§

(B) Relapse*‡: Total lung CFU 3 months after treatment with the Standard Regimen or PRS Regimen III for the period indicated

	**Standard Regimen**		**PRS Regimen III**		
**Mouse**	6 weeks	8 weeks	4 weeks	5 weeks	6 weeks
1	1.2×10^{5}	3.5×10^{3}	0	0	0
2	1.6×10^{4}	1.1×10^{4}	0	0	0
3	$> 1.2 \times 10^{8}$	9.2×10^{3}	0	0	0
4	1.2×10^{7}	$> 1.2 \times 10^{8}$	0	0	0
5	$> 2.5 \times 10^{7}$	$> 1.5 \times 10^{8}$	0	0	0
6	1.4×10^{6}	4.9×10^{5}	0	0	0
7	7.8×10^{6}	2.8×10^{5}	0	0	0
8	6.8×10^{4}	$> 1.2 \times 10^{8}$	0	0	0
9		6.7×10^{5}	0	0	0
10		9.7×10^{6}	0		0

*Starting six weeks after aerosol infection, C3HeB/FeJ mice (n = 4§ - 5 per group) were treated daily for 14 days or 5 days per week (Monday-Friday) for 3, 4, 5, 6 or 8 weeks.

†For the efficacy study, lung log_{10} CFU were determined three days after the last treatment. Data shown are mean ± SEM. n.d., not done

‡For the relapse study, mice were held for 3 months after the last treatment dose and then euthanized for assay of lung CFU. Data are total lung CFU for each animal.
