# Supplementary figures and images for: Ultra-rapid near universal TB drug regimen identified via parabolic response surface platform cures mice of both conventional and high susceptibility
Source: PLoS One. 2018 Nov 14;13(11):e0207469. doi: 10.1371/journal.pone.0207469 (PMC6235396; doi:10.1371/journal.pone.0207469)

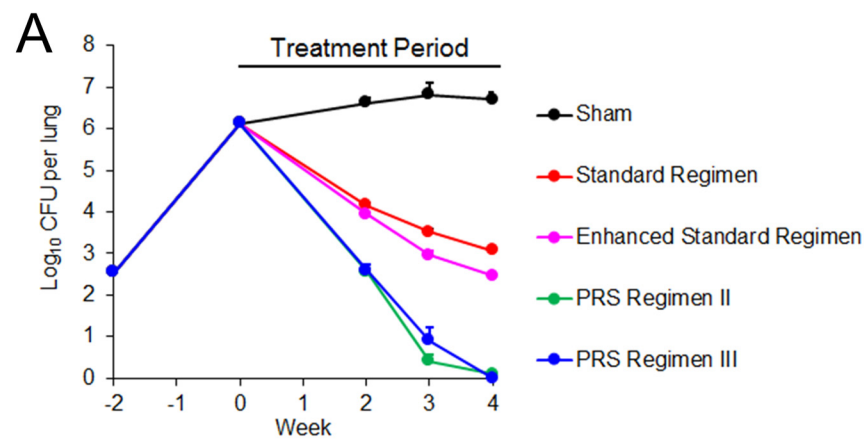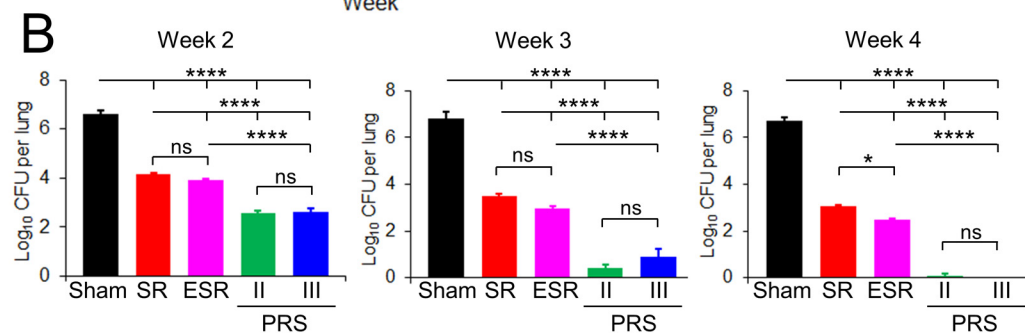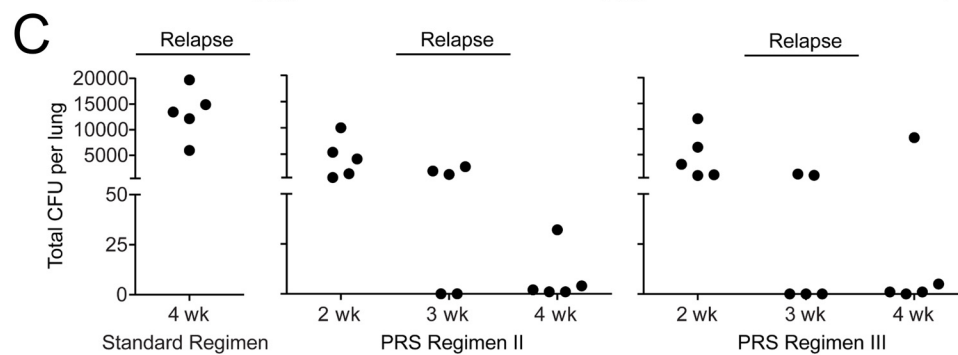

Supplement: S1 Fig — (A) M. tuberculosis burdens in the lung were determined over the course of infection and treatment period. (B) Lung burden of M. tuberculosis after treatment 5 days per week by oral gavage for 2, 3, and 4 weeks in sham-treated mice or mice treated with the Standard Regimen (SR), Enhanced Standard Regimen (ESR), PRS Regimen II (PRS II) or PRS Regimen III (PRS III). For mice with zero CFU in the lungs, a CFU count of 1 was assigned for graphing purposes. Two-way ANOVA with Tukey’s multiple comparison test was used in statistical analyses. **** p < 0.0001, * p < 0.05, ns, not significant (C) Number of M. tuberculosis organisms in the lung of each mouse 3 months after treatment cessation. (PDF) [file pone.0207469.s007.pdf]

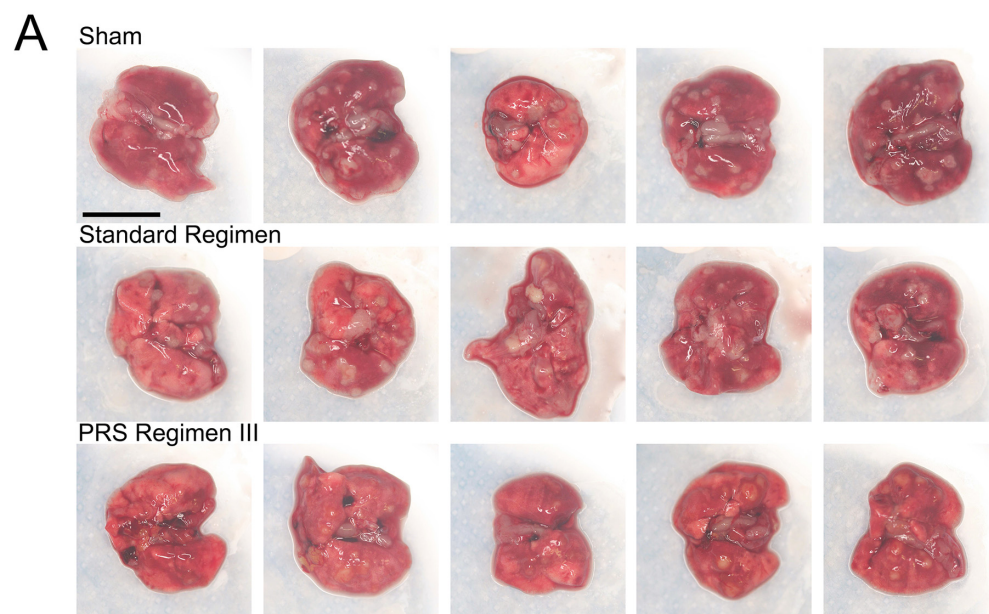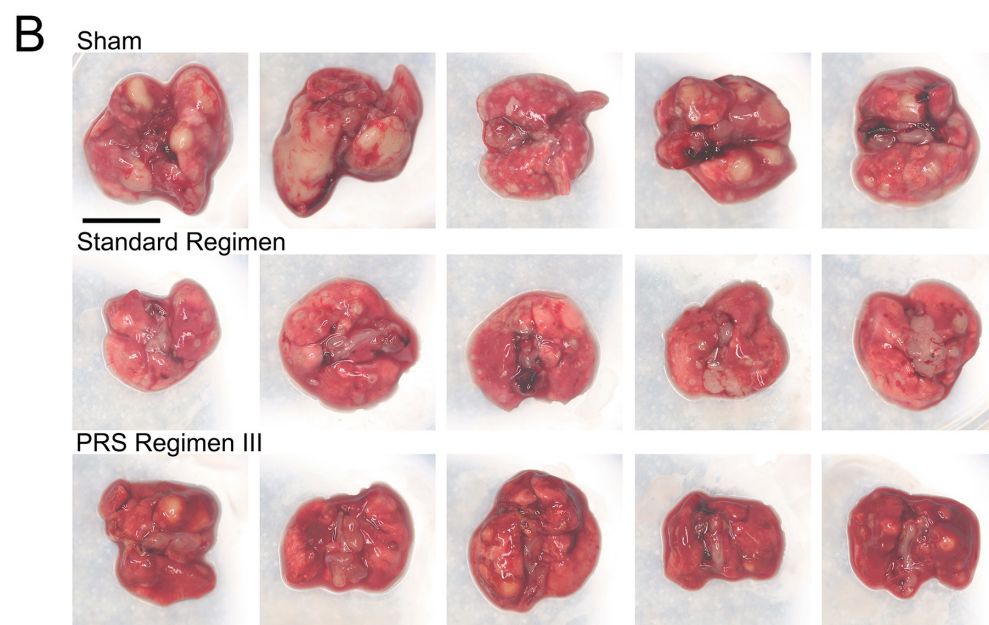

C

Sham

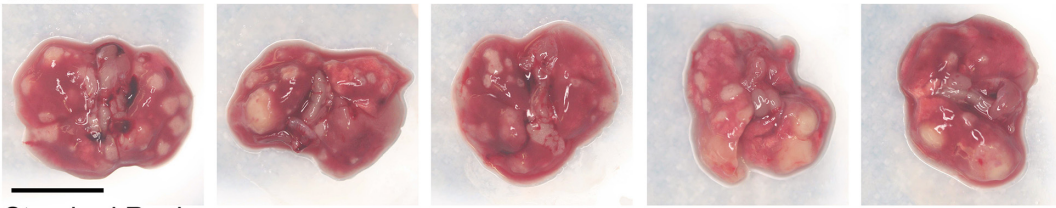

Standard Regimen

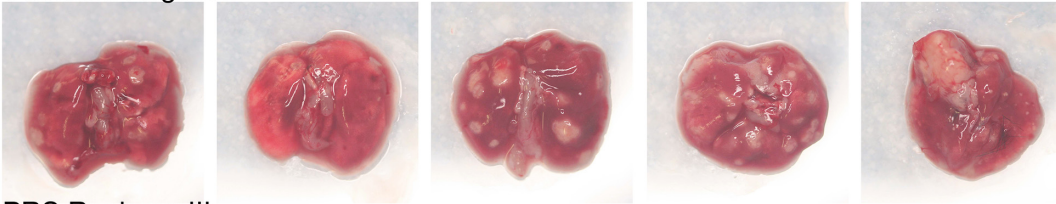

PRS Regimen III

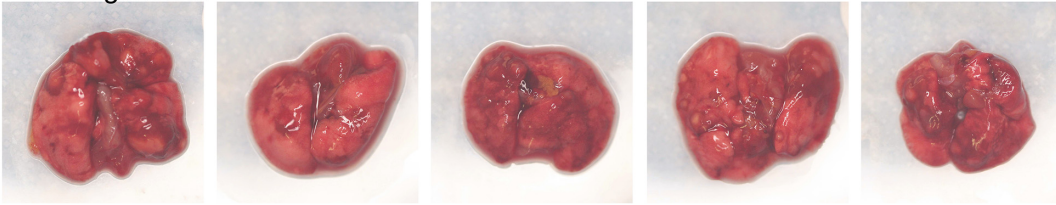

D

Sham

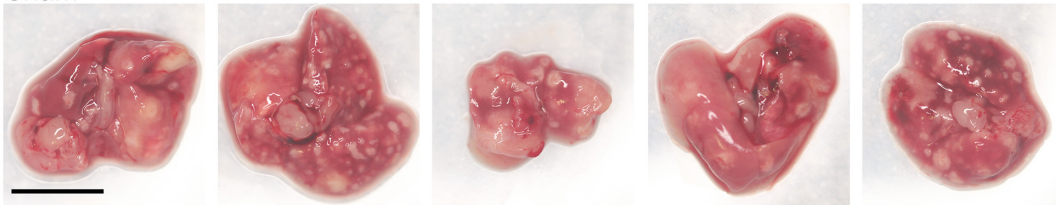

Standard Regimen

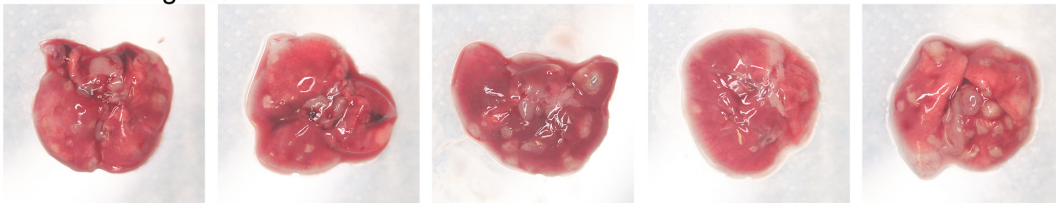

PRS Regimen III

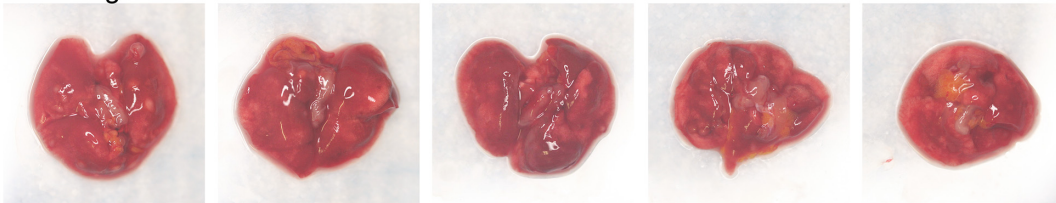

Supplement: S3 Fig — Mice were infected with M. tuberculosis by aerosol and starting six weeks later were sham-treated or treated with the Standard Regimen (INH/RIF/EMB/PZA at 25/10/100/150 mg/kg) or PRS Regimen III (CFZ/BDQ/SQ109/PZA at 25/30/25/450 mg/kg) 5 days per week for (A) 3 weeks, (B) 5 weeks, (C) 6 weeks, and (D) 8 weeks. The mice were then euthanized and their lungs and surface granulomas photographed. Scale bar (upper left panel), 1 cm. (PDF) [file pone.0207469.s009.pdf]

**A** PRS Regimen III

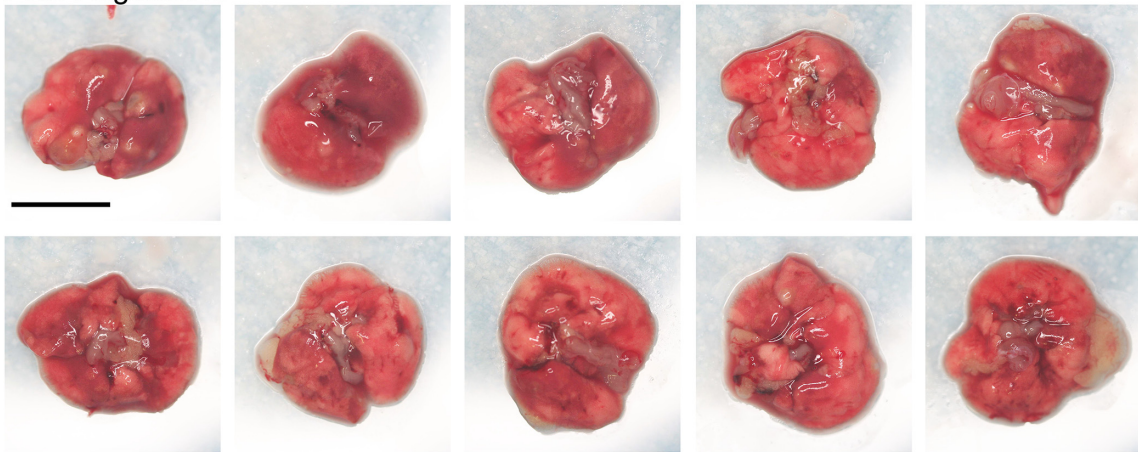

**B** PRS Regimen III

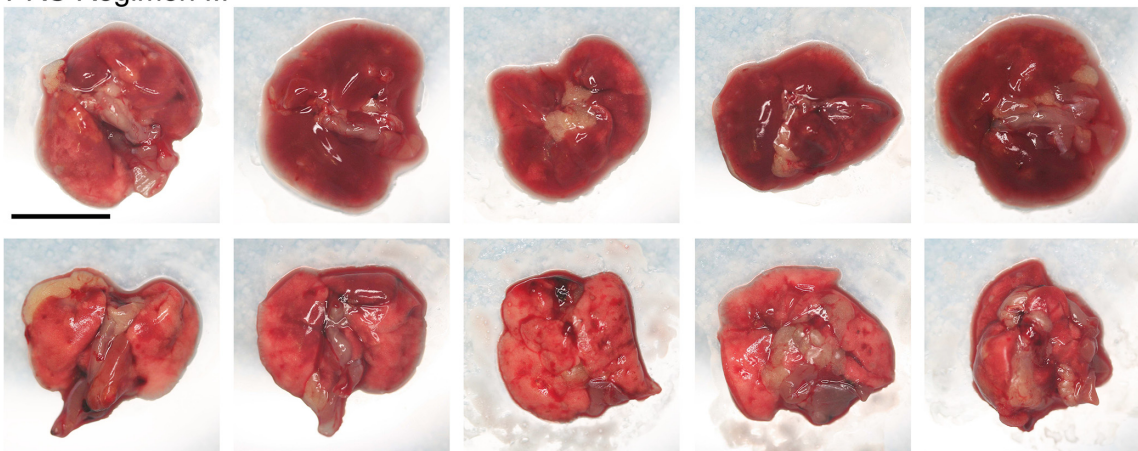

C Standard Regimen

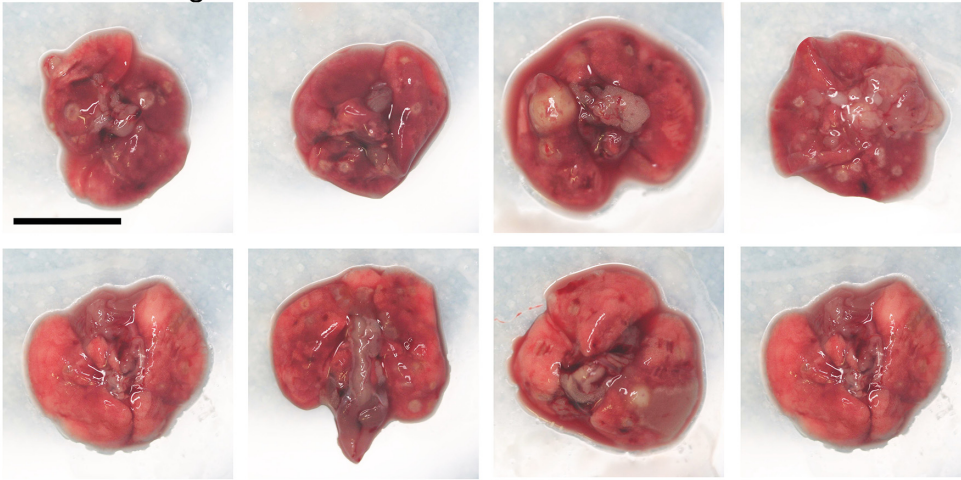

PRS Regimen III

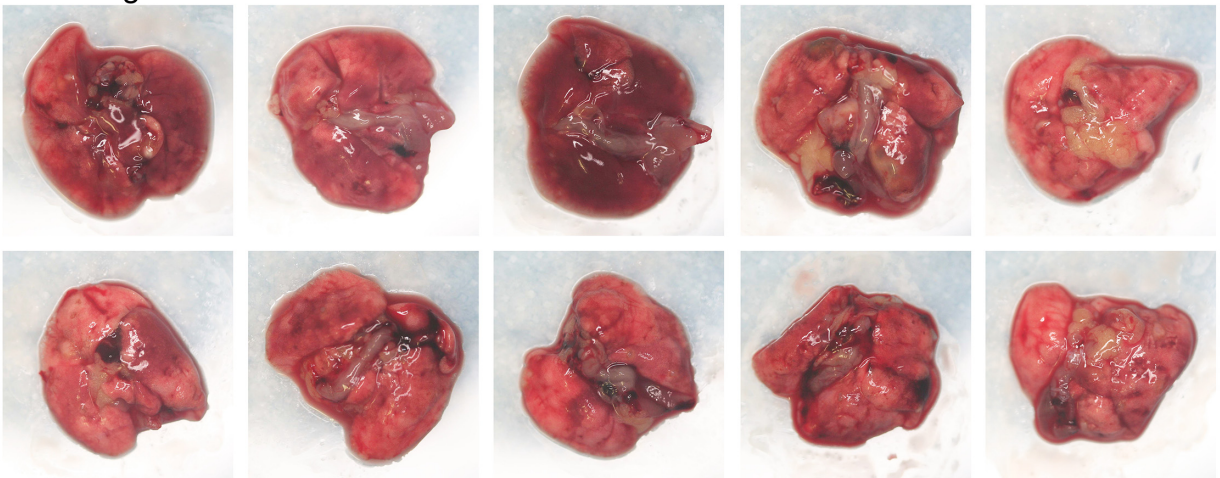

Supplement: S4 Fig — Photographs show the lungs and surface granulomas from mice 3 months after completion of treatment with the Standard Regimen (INH/RIF/EMB/PZA at 25/10/100/150 mg/kg) or PRS Regimen III (CFZ/BDQ/SQ109/PZA at 25/30/25/450 mg/kg) for (A) 4 weeks, (B) 5 weeks or (C) 6 weeks. Scale bar (upper left panel), 1 cm. (PDF) [file pone.0207469.s010.pdf]
