# Supplementary material for: Ultra-rapid near universal TB drug regimen identified via parabolic response surface platform cures mice of both conventional and high susceptibility
Source: PLoS One. 2018 Nov 14;13(11):e0207469. doi: 10.1371/journal.pone.0207469 (PMC6235396; doi:10.1371/journal.pone.0207469)
Supplement: S2 Fig — Mice were infected with M. tuberculosis by aerosol and starting two weeks later were sham-treated or treated with the Standard Regimen (INH/RIF/EMB/PZA at 25/10/100/150 mg/kg), PRS Regimen II (CFZ/BDQ/EMB/PZA at 25/30/100/450 mg/kg) or PRS Regimen III (CFZ/BDQ/SQ109/PZA at 25/30/25/450 mg/kg) 5 days per week for (A) 3 weeks, (B) 5 weeks or (C) 6 weeks. The mice were then euthanized and their lungs and surface granulomas photographed. Scale bar (upper left panel), 1 cm. (PDF) [file pone.0207469.s008.pdf]

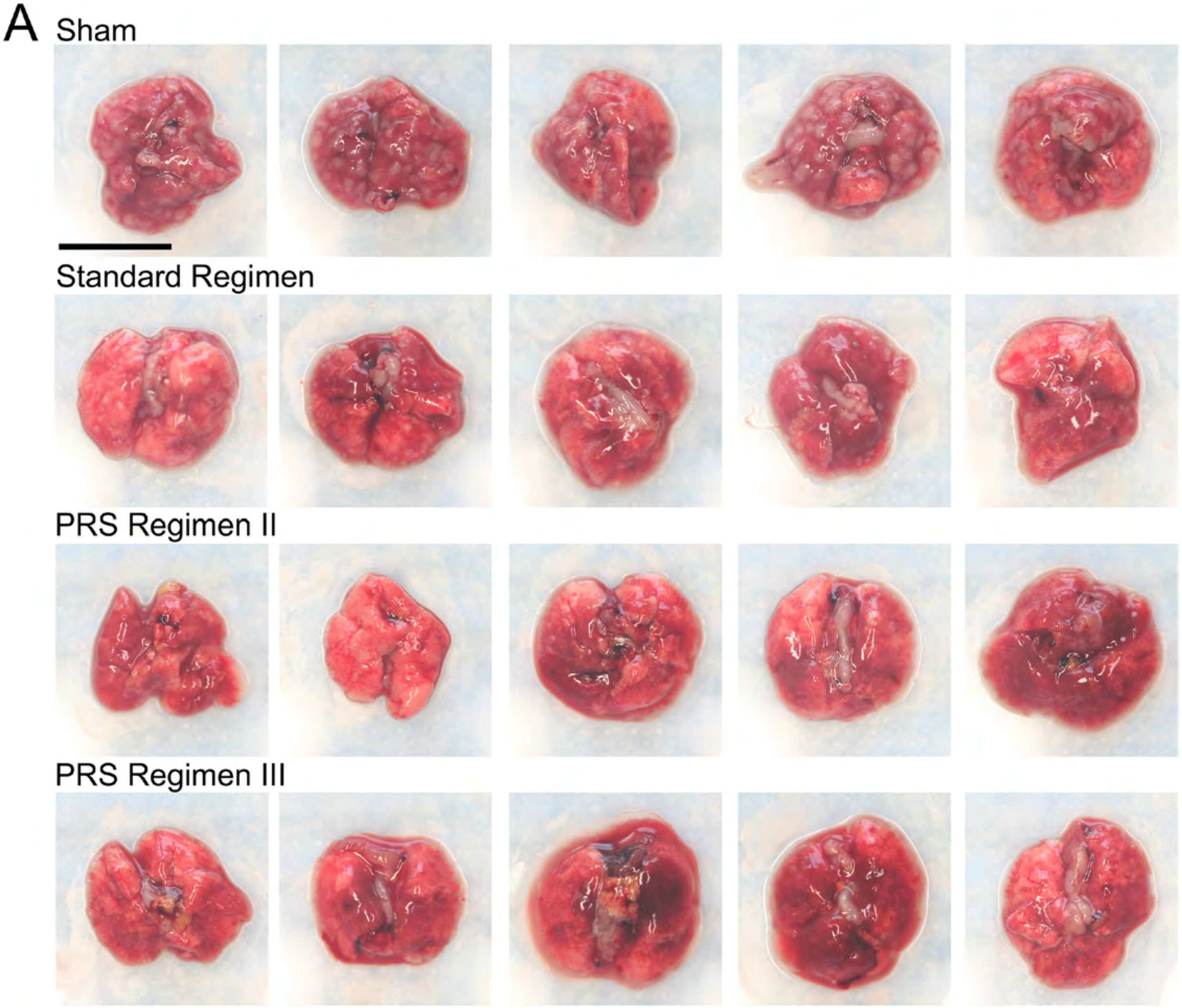
A
Sham
Standard Regimen
PRS Regimen II
PRS Regimen III

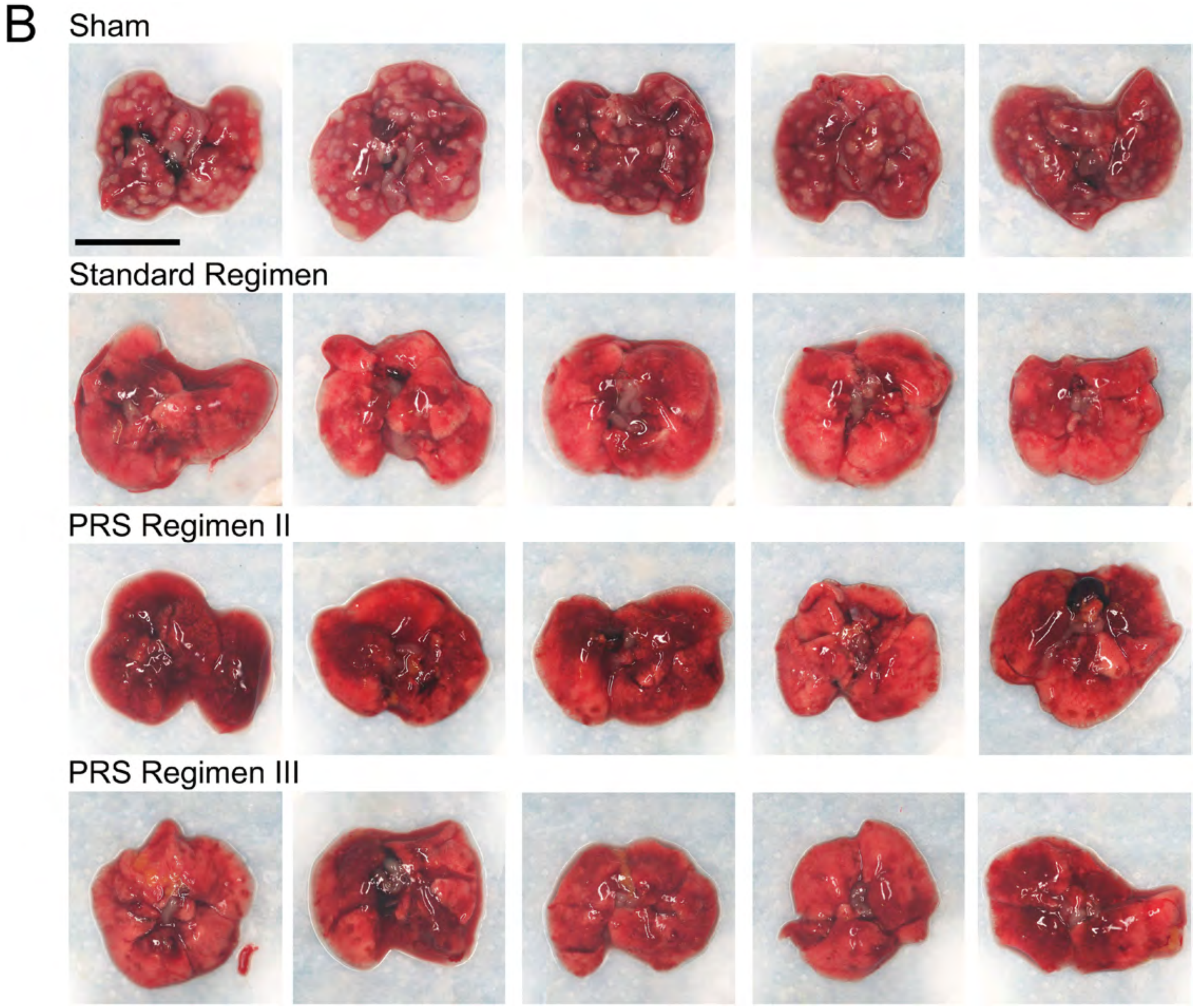
B
Sham
Standard Regimen
PRS Regimen II
PRS Regimen III

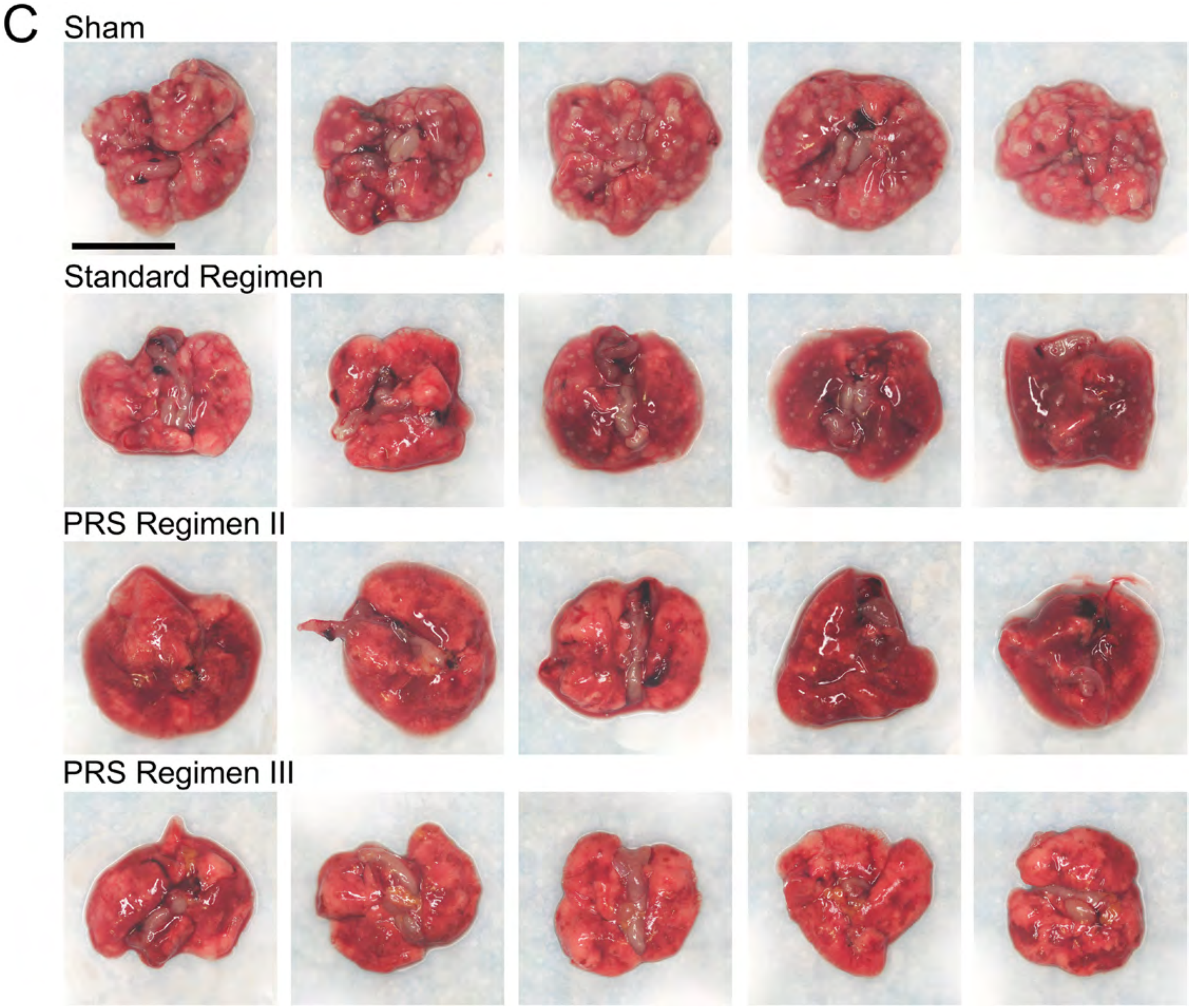
C
Sham
Standard Regimen
PRS Regimen II
PRS Regimen III
