# Supplementary material for: Ultra-rapid near universal TB drug regimen identified via parabolic response surface platform cures mice of both conventional and high susceptibility
Source: PLoS One. 2018 Nov 14;13(11):e0207469. doi: 10.1371/journal.pone.0207469 (PMC6235396; doi:10.1371/journal.pone.0207469)
Supplement: S1 Code — (PDF) [file pone.0207469.s011.pdf]

S1 Code. PRS Regimen III MATLAB code

```
%%%%%%% 1. LOAD DATA %%%%%%%

data = [

%Data for PRS Regimen III

 %CFZ  SQ109  BDQ   PZA  Output  Group

  25   2.78   5.6   450   1.39     %C

  25   2.78   50    50    0.75     %D

  25    25    5.6   50    3.21     %E

  25    25    50    450   0.23     %F

  25    25    50    50    0.61     %G

  25    25    5.6   450   1.63     %H

  25   2.78   50    450   0.33     %I

  25    25   16.7   150   1.05     %J

  25   8.25   50    150   0.65     %K

  25   8.25  16.7   450   0.54     %L

  25  16.75   50    450   0.54     %M

  25   8.25   50    450   0.36     %N

];

%%%%%%% 2. DEFINE INPUTS AND OUTPUTS FOR PRS MODEL %%%%%%%

X_o = data(1:12,1:4);

Y_o = data(1:12,5);


%%%%%%% 3. GENERATE PRS MODEL %%%%%%%

result =
LinearModel.stepwise(X_o,Y_o,'quadratic','ResponseVar','Log10CFU','PredictorVars',
{'CFZ','SQ109','BDQ','PZA'})

%%%%%%% 4. FIND CORRELATION %%%%%%%

correlation = corr(Y_o,result.Fitted)
```
